# Understanding soil factors in corrosion and conservation of buried bronze statuettes: insights for preservation strategies

**DOI:** 10.1038/s41598-024-69490-5

**Published:** 2024-08-20

**Authors:** Mohamed Abdelbar, Ashraf M. El-Shamy

**Affiliations:** 1 Conservation Department, Faculty of Archaeology, Damietta University, Damietta El-Gadeeda City, 34511 Damietta Governorate Egypt; 2 Physical Chemistry Department, Electrochemistry and Corrosion Laboratory, National Research Centre, El-Bohouth St. 33, Dokki, P.O. 12622, Giza, Egypt

**Keywords:** Corrosion conditions, Soil chemistry, Conservation approach, Bronze age, Burial condition, Electrochemical corrosion, Chemistry, Materials science

## Abstract

The findings reveal that soil constituents significantly affect the corrosion process. Moisture content and pH promote the formation of corrosion products, while high chloride concentrations accelerate corrosion. Conversely, high organic matter content inhibits corrosion by limiting oxygen diffusion to the metal surface. The effectiveness of conservation treatments, particularly wax or oil-based coatings, varied with soil conditions, showing reduced efficacy in soils with high chloride concentrations. This study underscores the importance of understanding soil constituents for developing effective corrosion and conservation strategies for buried bronze statuettes. The results offer valuable insights for customizing preservation approaches based on soil types. X-ray diffraction (XRD) analysis revealed that mineralogical compositions in soil significantly influence corrosion processes, providing critical insights for effective preservation strategies. pH measurements indicated varying soil acidity and alkalinity levels, crucial in determining corrosion rates and mechanisms, offering essential data for targeted preservation strategies. Additionally, the identification of brochantite and antlerite through Micro-Raman spectroscopy suggests a link to sulfur pollutants from the decomposition of organic matter by sulfate-reducing bacteria, highlighting the potential environmental impact of microbial activity in the soil ecosystem.

## Introduction

Buried bronze statuettes, revered relics of cultural heritage, face an insidious threat beneath the soil: corrosion. Understanding the intricate interplay between soil factors and the corrosion process is paramount to the preservation of these irreplaceable artifacts. This advanced introduction delves into the multifaceted realm of soil chemistry, microbiology, and environmental conditions that influence corrosion dynamics, offering profound insights for the development of preservation strategies^1–3^. The soil environment, often perceived as a silent antagonist, harbors a plethora of chemical species, microbial communities, and physical conditions that catalyze or mitigate corrosion processes. Chemical constituents such as moisture content, pH levels, salinity, and the presence of sulfides profoundly impact the corrosion kinetics of buried bronze statuettes. Furthermore, the intricate interactions between soil moisture and oxygen availability play a pivotal role in accelerating or retarding corrosion rates, shaping the fate of these artifacts over time^4–7^. Microbial communities residing within the soil matrix exhibit remarkable diversity and metabolic versatility, exerting significant influence on corrosion phenomena. Microbial-induced corrosion (MIC), driven by the metabolic activities of sulfate-reducing bacteria (SRB) and other anaerobic microorganisms, poses a formidable challenge to the preservation of buried bronze statuettes. Understanding the microbial ecology and biogeochemical pathways involved in MIC is essential for devising targeted conservation strategies to mitigate its deleterious effects^8^. Environmental factors, including climate variations, seasonal fluctuations, and anthropogenic activities, further complicate the corrosion landscape. Exposure to fluctuating environmental conditions can exacerbate corrosion processes, leading to accelerated deterioration of buried bronze statuettes. Moreover, human interventions, such as land-use changes, agricultural practices, and urban development, can inadvertently alter soil properties and exacerbate corrosion risks, necessitating a holistic approach to conservation planning^9–12^. In light of these complexities, elucidating the intricate nexus between soil factors and corrosion dynamics is imperative for the development of effective preservation strategies. By integrating insights from soil science, corrosion engineering, microbiology, and conservation science, researchers can devise tailored approaches to mitigate corrosion risks, prolong the longevity of buried bronze statuettes, and safeguard our cultural heritage for future generations. This advanced introduction sets the stage for a comprehensive exploration of soil-related corrosion mechanisms and conservation methodologies, offering profound implications for the preservation of these invaluable artifacts^13–16^. Buried beneath the earth’s surface, bronze statuettes silently endure a battle against a relentless adversary: corrosion. These cultural treasures, imbued with historical significance and artistic mastery, face the gradual decay wrought by environmental forces. Understanding the intricate dynamics of corrosion and implementing effective preservation strategies are imperative to safeguarding these irreplaceable artifacts. This advanced introduction embarks on a journey to unravel the complexities of corrosion in buried bronze statuettes, offering profound insights into preservation methodologies^17–20^. Corrosion, an omnipresent phenomenon driven by chemical reactions, transforms the surface of bronze statuettes over time. Beneath the protective veil of soil, a multitude of factors converge to initiate and propagate corrosion processes. Soil composition, moisture content, pH levels, and the presence of corrosive agents such as sulfides and chlorides exert profound influences on corrosion kinetics. Unraveling the intricate interplay between these soil factors and corrosion dynamics is essential to comprehending the underlying mechanisms dictating the fate of buried bronze statuettes^21–24^. Moreover, the microbial inhabitants of the soil biome, often overlooked but wielding significant influence, contribute to corrosion processes through microbial-induced corrosion (MIC). Sulfate-reducing bacteria (SRB) and other microbial species thrive in anaerobic environments, accelerating corrosion rates through metabolic activities. Understanding the microbial ecology of soil and its implications for corrosion dynamics is paramount to devising targeted conservation strategies that mitigate microbial-driven degradation^25,26^. In addition to natural environmental factors, anthropogenic activities, and climate variations further complicate the corrosion landscape. Urban development, agricultural practices, and industrial pollution can introduce contaminants into the soil, exacerbating corrosion risks. Climate fluctuations, seasonal changes, and exposure to extreme weather events pose additional challenges to preservation efforts, necessitating adaptive conservation methodologies^27^. As custodians of cultural heritage, conservationists and researchers confront the daunting task of preserving buried bronze statuettes for future generations. By unraveling the intricacies of corrosion mechanisms and soil interactions, they gain invaluable insights into the preservation challenges faced by these artifacts. Drawing upon interdisciplinary expertise in materials science, corrosion engineering, archaeology, and conservation science, researchers can devise holistic preservation strategies that mitigate corrosion risks, prolong the lifespan of bronze statuettes, and safeguard our cultural legacy for posterity^28,29^. In summary, this advanced introduction sets the stage for an in-depth exploration of corrosion and conservation methodologies for buried bronze statuettes. By delving into the complexities of corrosion processes and soil interactions, researchers embark on a transformative journey toward the preservation of these invaluable cultural artifacts, ensuring their enduring legacy for generations to come^30^. The primary objective is to comprehensively investigate the soil factors influencing corrosion processes in buried bronze statuettes. This includes analyzing soil composition, moisture content, pH levels, presence of corrosive agents, and microbial communities to understand their roles in corrosion dynamics^31^. Another objective is to elucidate the underlying corrosion mechanisms driven by soil factors. By understanding the chemical and microbial processes contributing to corrosion, the study aims to identify key pathways and interactions influencing the degradation of bronze statuettes^32^. The study seeks to develop innovative preservation strategies informed by the insights gained from soil analysis and corrosion mechanisms. These strategies aim to mitigate corrosion risks, prolong the lifespan of buried bronze statuettes, and enhance their preservation for future generations^33,34^. Additionally, the study aims to evaluate existing conservation techniques and assess their effectiveness in combating corrosion in buried bronze statuettes. By comparing different preservation approaches, the research aims to identify best practices and optimize conservation efforts^35,36^. The study adopts an interdisciplinary approach, integrating principles from soil science, corrosion engineering, microbiology, archaeology, and conservation science. This holistic approach allows for a comprehensive understanding of the complex interactions between soil factors, corrosion processes, and preservation strategies^37^. An innovative aspect of the research is the analysis of microbial-induced corrosion (MIC) in buried bronze statuettes. By investigating the microbial communities present in the soil environment and their impact on corrosion, the study sheds light on a previously overlooked aspect of degradation mechanisms. The study goes beyond the mere identification of corrosion factors and mechanisms by actively developing novel preservation strategies. By leveraging insights from soil analysis and corrosion mechanisms, the research aims to propose innovative conservation techniques tailored to the unique challenges of preserving buried bronze statuettes. The research emphasizes the practical applications of its findings by directly informing conservation practices and preservation efforts. By offering tangible strategies for mitigating corrosion risks and enhancing preservation outcomes, the study aims to make a meaningful impact on the field of cultural heritage conservation.

## Material and methods

### Materials

**Figure 1:**
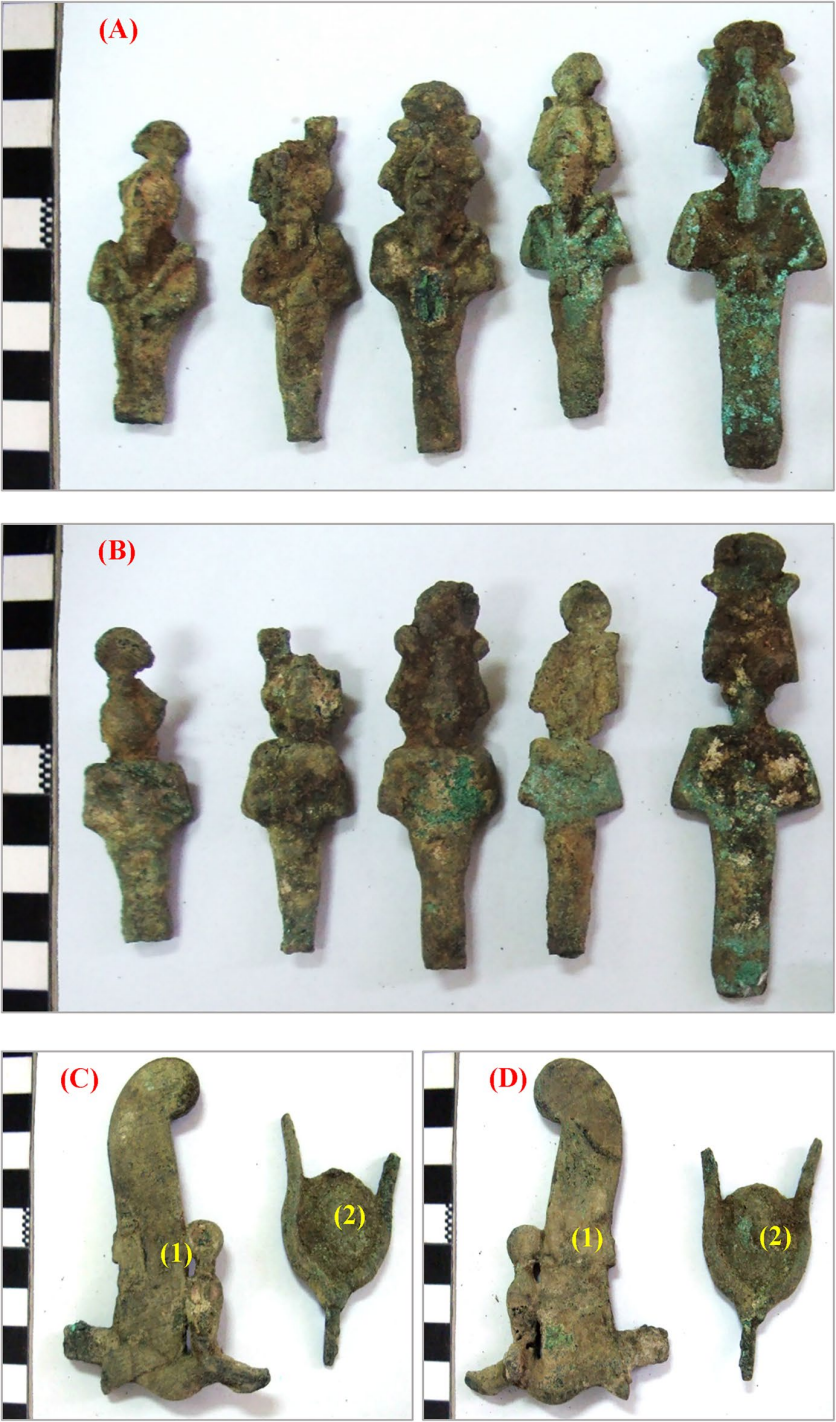
The Statuettes of the God Osiris—detailed views, (**A**, **C**) Front sides and (**B**, **D**) Back sides of the statuettes showcasing: Bronze Atef Crown’s feather (highlighted in both front and back views), Sun disk set between a pair of bull’s horns (visible in the front views).

The museum store at Kom Oshiem in El-Fayoum, Egypt, houses a collection of five small bronze statues of the god Osiris, a feather from a bronze Atef Crown, and a solar disc from a statue of the goddess Hathor. These artifacts, dating back to the Late Period (664-332 BC), depict Osiris in a standing position, with the statues varying in length from 6.75 to 8.9 cm. The feather from the Atef Crown, part of a larger figure of Osiris, features a ram’s horn at the base supporting a cobra, likely adorned with inlays and crowned with a solar disc, and measures approximately 8.3 cm in length. The bronze headdress, resembling a sun disk flanked by bull’s horns, likely belonged to a figure of Hathor and measures about 6 × 5.2 cm^38^. These statuettes were discovered in a deteriorated state at Ehnasya during the accidental construction of drinking water lines in 1956. Ehnasya, also known as Ahnas, is located about 70 miles south of Cairo and 12 miles from the Nile. It holds historical and mythological significance, dating back to the First Dynasty. The cultivation strip extending four miles between the town and the desert suggests it may have originally been situated on the desert’s edge. Currently, Ehnasya is within the prefecture of Beni Suef near the entrance to El-Fayoum Oasis. Bronze samples were selected for analysis to study their microstructure, alloy composition, and deterioration processes^39^. The statuettes of Osiris in Fig. 1 are significant artifacts related to a central figure in ancient Egyptian mythology and religion. We discuss the front and back views of these statuettes, along with the unique features of the Bronze Atef Crown’s feather and the sun disk headdress. The front views (A and C) provide insights into the iconography and symbolism of Osiris, often depicted as a mummified figure with a pharaoh’s beard and wearing the Atef Crown, representing the god of the afterlife, resurrection, and fertility. These statues emphasize his role as a divine ruler and judge of the dead, with meticulous craftsmanship reflecting the reverence attributed to him in ancient Egyptian culture^40^. The back views (B and D) offer additional details and perspectives, enriching our understanding of their craftsmanship and artistic expression. While the front views focus on Osiris’ iconic attributes and regal stance, the back views may reveal additional symbolic elements or inscriptions that enhance the narrative surrounding the deity. Including back views allows for a more comprehensive analysis of their design and symbolism. The Bronze Atef Crown’s feather (1) enhances the symbolic significance of these statuettes. The Atef Crown, associated with Osiris and other deities, typically features ostrich feathers, ram’s horns, and the sun disk. The ostrich feather symbolizes truth, justice, and cosmic order, aligning with Osiris’ role as a judge in the afterlife. The feather underscores Osiris’ divine authority and role in maintaining cosmic balance. The depiction of the sun disk set between bull’s horns (1) emphasizes Osiris’ association with solar symbolism and fertility. The sun disk symbolizes the sun god Ra, representing light, warmth, and regeneration, while the bull’s horns signify strength, power, and virility. This combination highlights Osiris’ role in the cycle of life, death, and rebirth, underscoring his multifaceted nature as a deity associated with fertility, renewal, and the afterlife. In conclusion, the Osiris statuettes in Fig. 1 offer valuable insights into ancient Egyptian religious beliefs, iconography, and craftsmanship. The front and back views provide a holistic perspective on Osiris’ portrayal, while the symbolic elements of the Bronze Atef Crown’s feather and the sun disk headdress enrich our understanding of his divine attributes and significance in ancient Egyptian mythology.

#### Physical appearance of corrosion products

The corrosion products on the statuettes are indicative of their prolonged burial and exposure to soil constituents. The green and blue patina is primarily composed of copper carbonate (malachite and azurite) and copper chloride compounds, which form due to the reaction of bronze with carbon dioxide and chloride ions in the soil. The crusty and powdery deposits are often the result of copper chloride (nantokite) converting to basic copper chlorides like atacamite and paratacamite, creating a rough and uneven surface. The dark patches may be attributed to the presence of sulfides and oxides, which form under low-oxygen conditions, indicating areas where organic matter decomposition by sulfate-reducing bacteria has occurred. A little bit reddish-brown spots are related to iron contamination from soil or groundwater can lead to iron oxide (rust) formation, adding another layer of complexity to the corrosion products. These corrosion products not only alter the physical appearance of the statuettes but also provide crucial information about the environmental conditions they were exposed to during burial, guiding effective conservation strategies.

**Figure 2:**
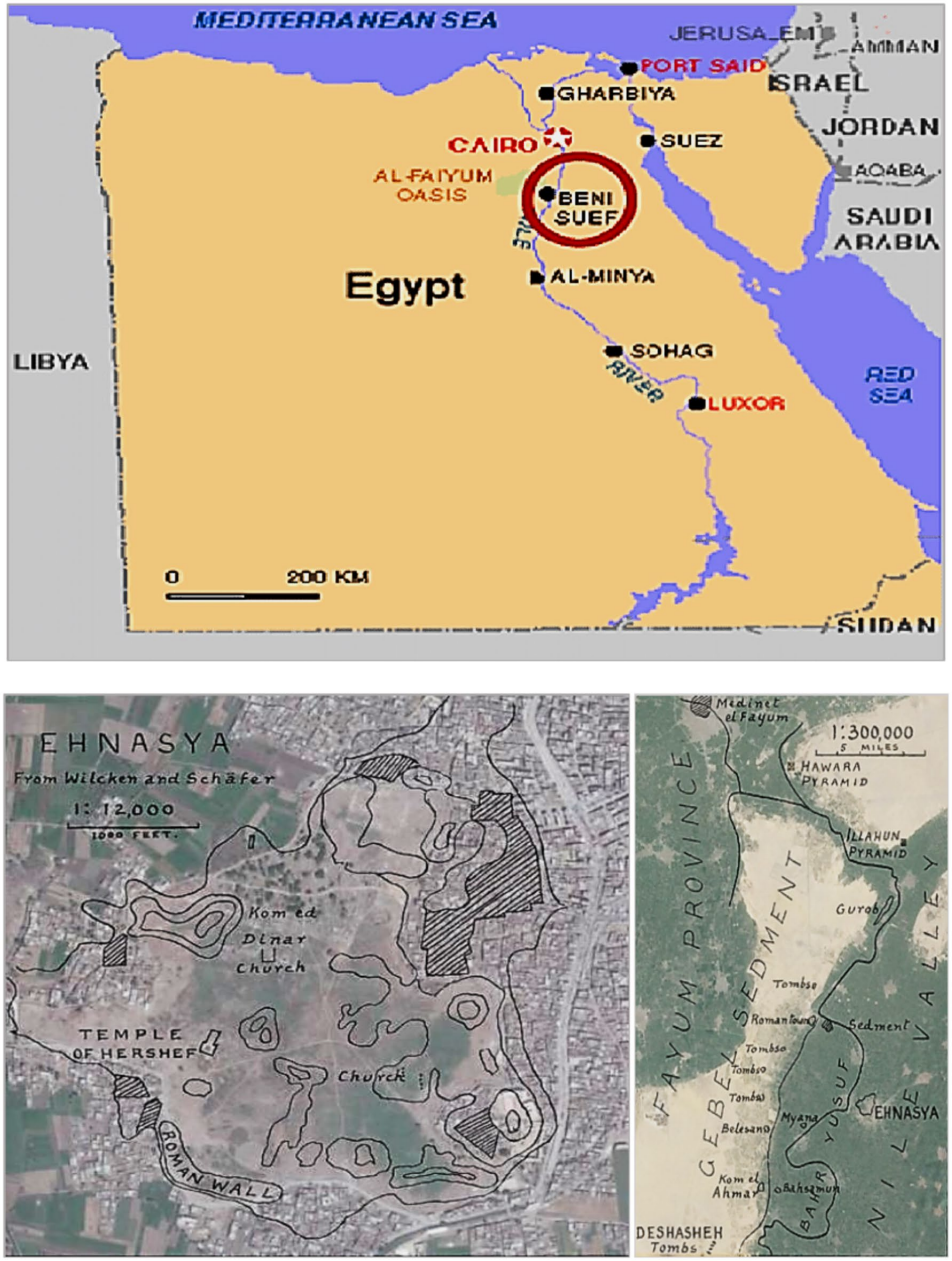
Unveiling the archaeological treasure trove mapping the location of ancient Ehnasya, the site of statuette excavation.

Figure 2 provides a geographical perspective on the location of ancient Ehnasya, the archaeological site where the statuettes depicted in Fig. 1 were excavated. Ehnasya, also known as Ahnas, holds significant historical and mythological importance in ancient Egyptian civilization. Located approximately 70 miles south of Cairo and 12 miles from the Nile, Ehnasya was known as Herakleopolis II Magna during the Roman era and Henensuten in ancient Egyptian times. Its strategic position along the Nile River made it a crucial religious and administrative center, facilitating trade, agriculture, and cultural exchange. The four-mile cultivation strip between Ehnasya and the surrounding desert landscape is believed to result from Nile soil covering the low desert terrain, suggesting the town may have initially been situated on the edge of the desert. Excavations at Ehnasya have yielded valuable insights into ancient Egyptian civilization, including artifacts, inscriptions, and architectural remains from various periods. The accidental discovery of the statuettes during the construction of drinking water lines in 1956 highlights the archaeological significance of Ehnasya and the importance of ongoing excavation and preservation efforts. Overall, Fig. 2 visually represents the archaeological treasure trove of ancient Ehnasya, emphasizing its rich history, cultural significance, and contribution to our understanding of ancient Egyptian civilization. It underscores the need for ongoing archaeological research and conservation efforts to preserve and interpret Ehnasya’s legacy for future generations.

### Methods

#### Sample extraction and performing

The samples were extracted and sampled from the bronze statuettes at specific locations to study their microstructure, alloy composition, and the nature of deterioration processes. Table 1 presents an illustration of how and where the sampling was performed.

**Table 1:** Comprehensive overview of bronze statuette sampling and analysis procedures.

Item	Description
Identification of sampling sites	The statuettes were carefully examined to identify appropriate sites for sampling. This involves selecting areas that are representative of the overall material while minimizing damage to the artifacts. Common areas for sampling include the base, edges, or areas with visible corrosion products or damage
Extraction procedure	A small drill or scalpel was used to extract minute samples from the identified sites. The tools and methods chosen ensure minimal impact on the integrity of the statuettes. Each sample was labeled and documented to keep track of its exact location and context
Preparation for analysis	The extracted samples were cleaned and prepared for various analyses. This could involve mounting the samples in resin, polishing, and preparing thin sections for microscopic examination. The samples were then subjected to techniques like optical microscopy, scanning electron microscopy (SEM), and energy-dispersive X-ray spectroscopy (EDS) to study their microstructure and composition
Location of sampling	The sampling was performed at Kom Oshiem in El-Fayoum, Egypt, where the artifacts are stored. The location ensures that the samples are extracted under controlled conditions and can be immediately prepared for analysis without transportation-related contamination or degradation

By following these steps, researchers ensured that the sampling was done carefully and systematically, allowing for detailed study of the bronze statuettes while preserving their historical and cultural integrity.

The degradation level was evaluated using a Rohs USB handheld digital microscope, which facilitated both visual and microscopic examinations. Sample preparation involved extracting a small piece from the statuettes and subjecting it to metallographic preparation^41^. This included polishing the sample with SiC broadsheet and 1/2 µm alumina suspension before examining it in both unetched and etched states^42^. For etching, an alcoholic FeCl_3_ solution was prepared by combining 240 ml of ethanol, 60 ml of hydrochloric acid, and 20 g of ferric chloride. Using an alcoholic FeCl_3_ (ferric chloride) solution is a practical approach for the cleaning and conservation of metal artifacts, particularly those made of bronze. This method involves using ferric chloride in an alcohol solution as an etchant and cleaner to remove corrosion products and surface contaminants from metal artifacts. Clean the artifact’s surface with a soft brush or cloth to remove loose dirt and debris. Submerge the artifact in the FeCl_3_ solution or apply the solution to the artifact’s surface using a brush or cotton swab. Ensure complete and even coverage of the solution on the corroded areas. Allow the solution to react with the corrosion products for a specified period, usually ranging from a few minutes to several hours, depending on the severity of corrosion and the solution’s concentration. Monitor the artifact closely during the treatment to avoid over-etching or damaging the metal. The FeCl_3_ solution will selectively dissolve the corrosion products, exposing the clean metal surface. After the desired cleaning effect is achieved, thoroughly rinse the artifact with clean alcohol to remove any residual FeCl_3_ solution and dissolved corrosion products. Depending on the treatment, a neutralizing agent (like sodium bicarbonate solution) may be used to neutralize any remaining acidic residues from the FeCl_3_ solution. Dry the artifact completely using a soft cloth or gentle air drying to prevent moisture-induced corrosion. After cleaning, apply a suitable protective coating, such as a wax or resin-based coating, to the artifact’s surface. This helps create a barrier against moisture and environmental contaminants, protecting the artifact from future corrosion. The FeCl_3_ solution provides a controlled method of removing corrosion products without extensive mechanical abrasion, which can be particularly beneficial for delicate or intricate artifacts. The solution is effective in selectively etching away corrosion layers while preserving the underlying metal, allowing for the conservation of fine details and inscriptions on the artifact. The alcohol base of the solution can help in controlling microbial growth on the artifact’s surface, which may contribute to corrosion. By effectively cleaning and stabilizing the artifact, the FeCl_3_ solution helps enhance the artifact’s long-term preservation and display value. This approach is widely used in conservation laboratories for the treatment of bronze and other metal artifacts, offering a balance between effectiveness and the preservation of the artifact’s integrity.

Surface morphology analysis of the alloy used in statuette production was conducted using the Leco L31 metallographic microscope^43^. The Inspect S50 Scanning Electron Microscope from FEI company (SEM) with energy dispersive spectroscopy (EDS) was utilized to examine a sample with the same cross-section to determine the mineral composition on the alloy surface. Furthermore, portable X-ray fluorescence (XRF) spectroscopy (Bruker S1 TITAN and TRACER 5i Handheld XRF) was employed to investigate the elemental chemical arrangement of the bronze alloy. X-ray diffraction analysis (XRD) was carried out using a D8 advanced X-ray diffractometer from Bruker in Germany to identify deterioration results and soil mineral composition^44,45^. Micro-Raman spectroscopy, a non-destructive analytical technique, was also employed. This technique is advantageous as it allows for direct examination without altering the sample, making it particularly suitable for studying valuable or sensitive artifacts. It enables in-situ analysis, facilitating the study of samples in their natural environment or real-world conditions, which is crucial in fields like archaeology, art conservation, and environmental science^46^. Additionally, Micro-Raman spectroscopy offers high spatial resolution, enabling examination at the micron scale, which is beneficial for analyzing heterogeneous materials or specific sample features. The technique excels in identifying chemical compounds and materials, leveraging the unique Raman spectra of each compound. Despite its strengths, Micro-Raman spectroscopy faces challenges such as fluorescence interference, especially in complex or organic materials, and limited sensitivity to certain types of materials with weak Raman scattering^47^. In conclusion, Micro-Raman spectroscopy is a versatile and invaluable tool for materials analysis, with applications across scientific disciplines. As technology progresses and new techniques emerge, it is expected to continue playing a significant role in advancing our understanding of various materials and their properties.

### Experiments of corrosion

#### Weight loss measurement

Corrosion, a natural phenomenon, occurs when metals are exposed to environmental elements such as moisture, oxygen, and acidic substances. This process poses significant challenges to the safety and efficiency of industrial equipment, infrastructure, and metal structures. Weight loss measurement emerges as a common technique for corrosion monitoring, involving the weighing of metal specimens before and after exposure to corrosive agents. The difference in weight offers an estimate of the metal loss attributable to corrosion^48^. Despite its simplicity and cost-effectiveness, weight loss measurement has inherent limitations. Notably, it fails to provide insights into the specific location and type of corrosion on the metal surface. Moreover, accurately measuring corrosion rates, particularly in cases of slow corrosion, proves challenging. Nonetheless, weight loss measurement remains widely employed across industries for corrosion monitoring. Its utility extends to assessing the efficacy of corrosion inhibitors and coatings, as well as determining the service life of metal structures^49^. Furthermore, data gleaned from weight loss measurements can inform the development of predictive models, enabling the implementation of proactive maintenance and repair strategies.

#### Electrochemical measurements

Corrosion represents a natural process wherein metals degrade as a result of their interaction with the surrounding environment, ultimately leading to material loss, structural damage, and eventual failure. Electrochemical measurements serve as a widely employed technique for monitoring and analyzing the corrosion behavior of metals across diverse industrial and environmental contexts. This discussion delves into the fundamentals of electrochemical corrosion monitoring and its practical implications. Electrochemical corrosion monitoring relies on the fundamental principles of electrochemistry, which entail the exchange of electrons between the metal and the surrounding electrolyte. The setup for electrochemical corrosion monitoring typically includes a metal electrode, a reference electrode, and a counter electrode immersed in an electrolytic solution. The potential difference between the metal electrode and the reference electrode, known as the corrosion potential, furnishes insights into the metal’s corrosion behavior. Common electrochemical measurements utilized for corrosion monitoring encompass polarization resistance, electrochemical impedance spectroscopy (EIS), and potentiodynamic polarization. Polarization resistance quantifies the rate of charge transfer at the metal-electrolyte interface, which correlates with the corrosion rate. EIS characterizes the impedance of the metal-electrolyte interface across a spectrum of frequencies, thereby elucidating the electrical properties of corrosion products. Potentiodynamic polarization assesses the current and potential response of the metal electrode to controlled potential scans, revealing both passive and active corrosion behavior. In practical applications, electrochemical corrosion monitoring finds utility across various industries. In the oil and gas sector, where equipment is subjected to harsh environments prone to severe corrosion, monitoring corrosion behavior enables timely detection and prevention of corrosion-related failures, thereby reducing maintenance costs and prolonging equipment lifespan. Similarly, in the aerospace and automotive industries, electrochemical corrosion monitoring aids in evaluating the corrosion resistance of components and coatings, averting potential catastrophic failures. In conclusion, electrochemical measurements offer a valuable means for monitoring and analyzing the corrosion behavior of metals in diverse industrial and environmental settings. By elucidating potential and current responses at the metal-electrolyte interface, these measurements furnish critical insights into corrosion behavior, facilitating proactive measures to mitigate failures, minimize maintenance expenses, and enhance the longevity of metal structures and equipment.

The electrochemical procedures, including open circuit potential (OCP), polarization curves, and impedance measurements, were conducted on the statues to assess their corrosion behavior. These techniques provide valuable insights into the electrochemical properties and the corrosion resistance of the bronze material. OCP measurements were performed to monitor the potential of the bronze surface without applying any external current. This helps in understanding the natural corrosion potential of the material in its environment. Polarization tests involved applying a varying external voltage to the bronze surface and measuring the resulting current. This technique helps in determining the corrosion rate and identifying the different electrochemical reactions occurring on the surface. Electrochemical impedance spectroscopy (EIS) was used to measure the impedance of the bronze surface over a range of frequencies. This method provides information about the resistive and capacitive behavior of the corrosion layers and helps in modeling the corrosion process. The electrolyte used for these studies typically mimicked the environmental conditions the statues were exposed to, such as a solution containing chloride ions to simulate marine or soil conditions. A common electrolyte used in such studies is a 3.5% NaCl solution, which represents the presence of chlorides that can accelerate corrosion. The practical approach of these electrochemical studies is to gain a deeper understanding of the corrosion mechanisms affecting the bronze statues. By simulating environmental conditions and using electrochemical techniques, we can identify the primary factors contributing to corrosion, evaluate the effectiveness of conservation treatments, develop strategies to mitigate future corrosion and provide recommendations for the optimal environmental conditions in museums to preserve the statues. These studies ultimately help in preserving historical artifacts by informing conservation practices and ensuring the longevity of the statues in museum environments.

#### Micro-Raman spectroscopy

Micro-Raman spectroscopy is a non-destructive technique used to analyze the vibrational modes of molecules and crystals in a material, providing insights into its chemical composition, crystal structure, and molecular bonding. A monochromatic laser beam, typically in the visible or near-infrared range, interacts with the sample, causing molecules and crystals to vibrate and change energy levels through inelastic scattering. The scattered light is then analyzed by a spectrometer, producing a Raman spectrum that reveals intensity peaks corresponding to specific vibrational modes. This method is essential in fields such as materials science, chemistry, and biology for characterizing samples ranging from microscopic particles to bulk materials^50–52^. For bronze patina analysis, Micro-Raman spectroscopy offers non-destructive, in situ analysis capabilities. Utilizing a JASCO NRS-450 coupled with a Leica optical microscope featuring 20 × objectives, the technique employed a laser with a wavelength of 531.95 nm and a 900 l/mm grating, achieving a spectral resolution of 28.54 cm^−1^ or 2.32 cm^−1^/pixel. Laser power was optimized at 7.2 mW to analyze various points on the patina surface, ensuring precise characterization while minimizing interference from adjacent layers^53,54^.

## Results and discussion

### Corrosion parameters results

Table 2 summarizes the key parameters affecting the corrosion and conservation of buried bronze statuettes, including moisture content, pH, chloride concentration, organic matter, and microbial induced corrosion (MIC). It also addresses the effectiveness of various preservation and conservation treatments under different soil conditions. Moisture content is a critical factor influencing the corrosion of buried bronze statuettes. High moisture levels in the soil facilitate electrochemical reactions that accelerate corrosion. Although the experimental results did not explicitly provide moisture content data, understanding its impact is essential for developing effective conservation strategies. Future studies should aim to include precise moisture content measurements. Improved moisture barriers and effective drainage systems can help mitigate moisture-induced corrosion. The pH level of the soil plays a significant role in the corrosion rates and mechanisms of bronze statuettes. Soils with varying levels of acidity or alkalinity can either accelerate or inhibit corrosion. The experimental results indicated varying pH levels, highlighting the importance of monitoring soil pH. Preservation strategies should include treatments that stabilize the soil pH to optimal levels for minimizing corrosion, such as the application of pH-adjusting agents. Chloride ions in the soil are known to accelerate the corrosion process. The experimental findings confirmed that elevated chloride concentrations significantly contribute to the corrosion of bronze statuettes. To combat this, the application of chloride-resistant coatings and barrier layers is recommended. These treatments can protect the metal surface from the aggressive action of chloride ions, extending the lifespan of the statuettes. Organic matter in the soil can have a dual effect on corrosion. High organic matter content was found to inhibit corrosion by limiting oxygen diffusion to the metal surface. However, the decomposition of organic matter can also produce sulfur compounds that may contribute to corrosion. The study identified brochantite and antlerite, linking their presence to sulfur pollutants from the decomposition of organic matter by sulfate-reducing bacteria. Utilizing organic coatings and treatments that protect the metal while allowing limited oxygen diffusion can be an effective conservation strategy. MIC, caused by the activity of microorganisms in the soil, can either accelerate or inhibit corrosion. The identification of specific compounds like brochantite and antlerite suggests a link to sulfur pollutants from microbial activity. This highlights the potential environmental impact of microorganisms on the corrosion process. Implementing biocidal treatments to control microbial activity in the soil can help manage MIC and protect the bronze statuettes from microbial corrosion. The effectiveness of conservation treatments, particularly wax or oil-based coatings, was found to vary with soil conditions. These treatments showed reduced efficacy in soils with high chloride concentrations. This underscores the need for tailored conservation approaches based on specific soil conditions. Conservation strategies should be developed to address the unique challenges posed by different soil types, ensuring optimal protection for the buried bronze statuettes. The impact of soil constituents on the corrosion of buried bronze statuettes is crucial for developing effective preservation strategies. By considering the effects of moisture content, pH, chloride concentration, organic matter, and microbial activity, and tailoring conservation treatments to specific soil conditions, the longevity and integrity of these cultural artifacts can be significantly enhanced.

**Table 2:** Key parameters influencing corrosion and preservation strategies for buried bronze statuettes.

Parameter	Description	Impact on corrosion	Experimental results	Preservation, conservation strategies
Moisture Content	Amount of water present in the soil	High moisture promotes corrosion	Higher corrosion results at 40% moisture content	Improved moisture barriers and drainage systems
pH	Measure of soil acidity or alkalinity	Varies corrosion rates and mechanisms	pH measurements indicated varying levels of acidity and alkalinity, influencing corrosion rates	Use of pH-adjusting treatments to stabilize soil pH
Chloride Concentration	Amount of chloride ions in the soil	Accelerates corrosion	Elevated chloride concentrations were identified as accelerators of corrosion	Application of chloride-resistant coatings and barrier layers
Organic matter	Decomposed plant and animal residues in the soil	Inhibits corrosion by limiting oxygen diffusion	High organic matter content was found to inhibit corrosion by limiting oxygen diffusion to the metal surface	Utilization of organic coatings and treatments to protect the metal
Microbial induced corrosion (MIC)	Corrosion caused by the activity of microorganisms in the soil	Can accelerate or inhibit corrosion	Specific compounds like brochantite and antlerite were identified, linking to sulfur pollutants from sulfate-reducing bacteria	Implementation of biocidal treatments to control microbial activity
Conservation treatments	Techniques used to preserve and protect the statuettes	Effectiveness varies with soil conditions	Wax or oil-based coatings showed reduced efficacy in soils with high chloride concentrations	Tailored conservation treatments based on specific soil conditions

#### Surface features and analysis of corrosion products

The statuettes were created using the lost wax process as a single unit. They have a thick layer of soil deposits on their surface, which is accompanied by a greenish corrosion product that varies in intensity between shades of pale and dark green. Some of the statuettes have signs of corrosion, while others still have a significant amount of metal at their core. By using a portable digital microscope, it was discovered that certain areas of the statuettes, such as the head, uraeus, crook, and flail, have been eroded and obscured by the layers of corrosion. There are also sections of the crown and feet that are missing, which can be attributed to the severe corrosion that occurred during the burial process.

#### Alloy microstructure

Leaded bronze alloys are a type of bronze that contains a small percentage of lead in addition to copper and tin. The addition of lead to the bronze alloy improves its machinability and wear resistance. The microstructure of leaded bronze alloys consists of alpha phase. In a professional scientific illustration of the microstructure of leaded bronze alloy, the alpha phase can be visualized using a combination of optical and electron microscopy. The alpha phase is characterized by a copper-rich matrix with small tin-rich particles. Under optical microscopy, the alpha phase appears darker due to its higher copper content. The small tin-rich particles in the alpha phase can be seen as dark spots distributed throughout the copper-rich matrix^55^. Under electron microscopy, the microstructure of the leaded bronze alloy can be further explored in greater detail. The fine structure of the alpha phase can be seen, and the distribution of the lead particles can also be visualized. The lead particles can be observed as small dark spots within the copper-rich alpha phase. In summary, a professional scientific illustration of the microstructure of leaded bronze alloy would show the alpha phase, as well as the lead particles distributed within the alpha phase. Optical and electron microscopy can be used to visualize the different phases and provide a detailed understanding of the microstructure of the alloy^56^.

**Figure 3:**
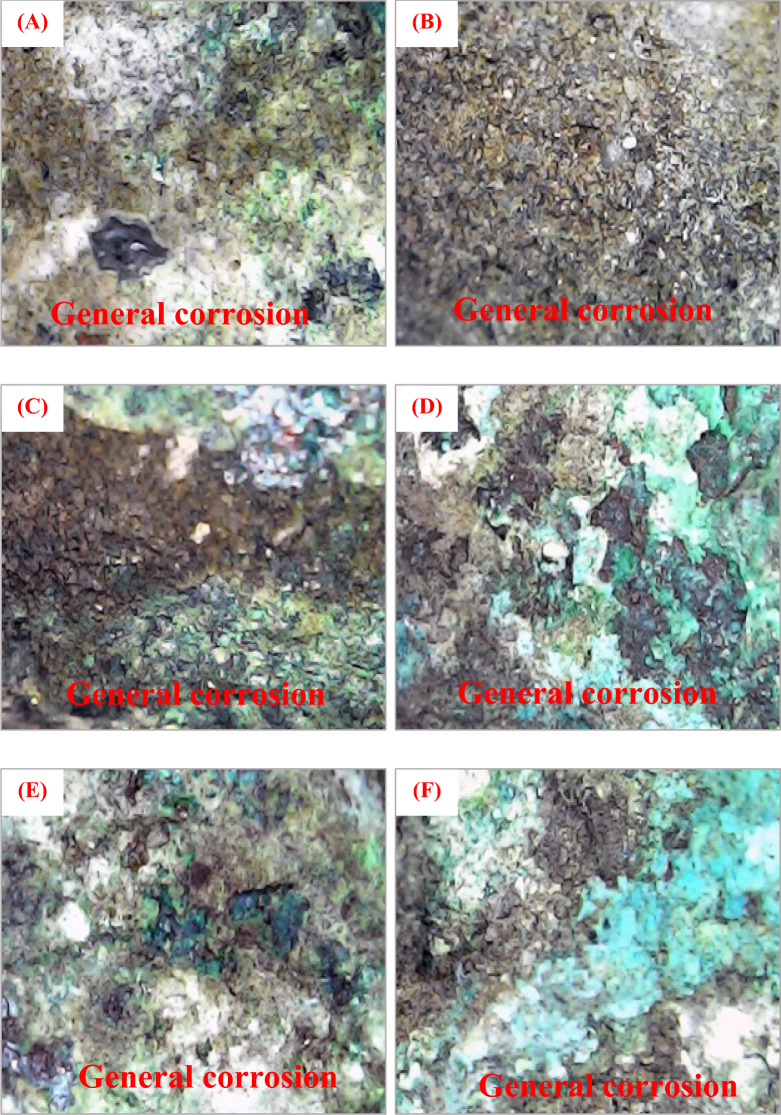
Microscopic images show (**A**–**F**) green and pale green corrosion incorporated with soil deposits covering the surface.

Figure 3 provides a set of microscopic images that offer valuable insights into the corrosion patterns observed on the surface of the bronze statuettes after burial in different soil types. These images vividly display the presence of green and pale green corrosion products that have formed and spread across the statuette surfaces, all of which are incorporated within soil deposits. The varying shades of green observed in these images are indicative of different corrosion products. These products are frequently linked to the interaction between the bronze alloy and environmental elements, such as moisture, oxygen, and certain ions found in the soil. The corrosion of bronze generally results in the creation of copper-based compounds like brochantite and antlerite, which are characterized by their greenish color. The intensity of this green coloration can act as a measure of the extent of corrosion. The presence of pale green areas suggests ongoing or less advanced corrosion, while darker green regions may indicate more advanced and extensive corrosion. The incorporation of soil deposits over the surface of the statuettes is another critical observation from these images. This phenomenon highlights the complex interaction between the bronze surface and the surrounding soil. The deposition of soil particles on the bronze surface can have a multifaceted impact on the corrosion process. On the one hand, it can create a physical barrier that limits exposure to environmental factors, potentially slowing down the corrosion rate. On the other hand, the soil itself can contain corrosive agents, such as chlorides or sulfides, which, when in contact with the bronze surface, may accelerate corrosion. These microscopic images underscore the importance of studying not only the corrosion products themselves but also their distribution patterns and the influence of soil interactions. Understanding the specific compounds formed and their spatial distribution can provide valuable information about the corrosion mechanisms at play. In summary, Fig. 3 reveals the visual evidence of corrosion products with varying degrees of advancement, as well as the intriguing interplay between these products and the soil deposits covering the bronze statuettes. This information contributes to a more comprehensive understanding of the corrosion processes affecting these cultural artifacts in different soil environments.

**Figure 4:**
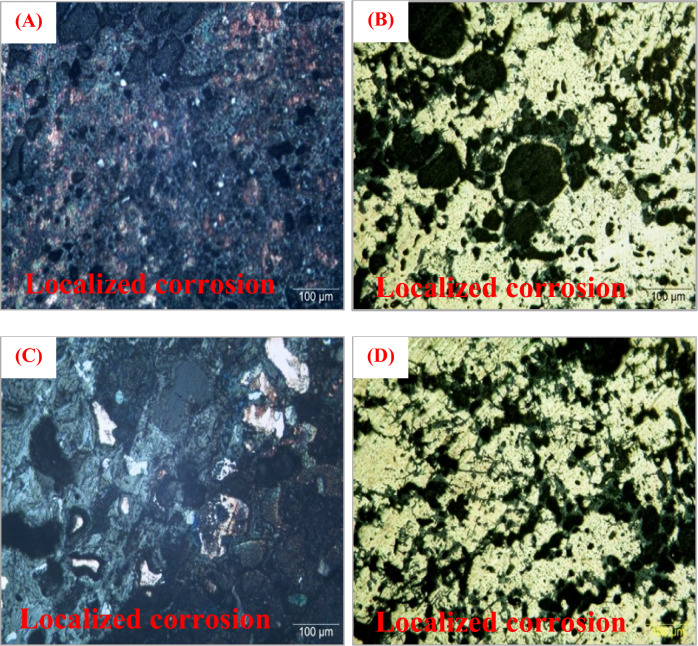
Presents the metallographic images (**A**, **B**) show the alloy microstructure of the statuettes of the un-etched sample and etched sample and (**C**, **D**) show the alloy microstructure of the statuettes of the un-etched sample and an etched sample of the plume.

Figure 4 provides metallographic images that are crucial for the examination of the alloy microstructure of the bronze statuettes. This microstructural analysis is particularly important for understanding the material composition and potential changes that occur because of burial in different soil environments. In the images, we observe both un-etched and etched samples of the bronze statuettes. The distinction between un-etched and etched samples is a standard practice in metallography, used to reveal different features of the microstructure. Etching, in this context, involves applying a chemical reagent to the sample, which selectively reacts with certain microstructural constituents, making them more visible under the microscope. (A-B) Un-etched sample and etched sample of the statuette. These images are particularly valuable for comparing the microstructure of the bronze alloy in its natural state (un-etched) with the same alloy after etching. The un-etched sample (A) shows the as-received microstructure, providing insight into the initial condition of the alloy. In this image, we can observe the grain boundaries and any inherent features in the alloy structure. The etched sample (B) highlights certain microstructural details more clearly by darkening certain features, revealing characteristics that may not be as apparent in the un-etched sample. (C-D) Un-etched sample and etched sample of the plume, Similar to (A-B), these images focus on the plume section of the bronze statuette. The un-etched sample (C) displays the natural microstructure of this specific region. Etching the sample (D) enhances the visibility of microstructural details, enabling a more comprehensive analysis of the plume’s alloy composition. These metallographic images are a valuable tool for researchers studying the effects of burial and corrosion on the bronze alloy. By comparing the un-etched and etched samples, scientists can identify changes in the microstructure that may be indicative of corrosion, material degradation, or chemical interactions with the burial environment. Furthermore, these images can aid in assessing the effectiveness of conservation and restoration efforts. If the etched samples show significant alterations in the microstructure, it may suggest the need for specific conservation strategies to address these changes and preserve the statuettes effectively. In summary, Fig. 4’s metallographic images offer a closer look at the alloy microstructure of the bronze statuettes, providing a foundation for understanding how burial and corrosion impact the material composition and structural integrity of these cultural artifacts.

**Figure 5:**
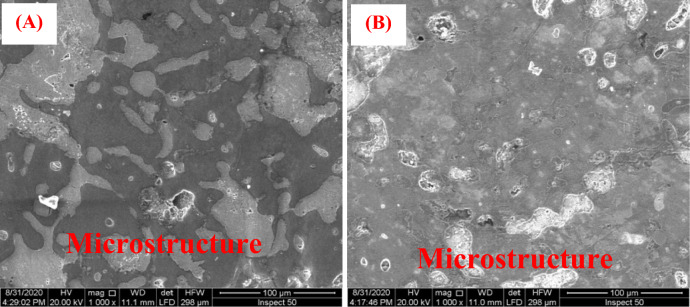
Presents the SEM image showing the alloy microstructure of (**A**) the plume and (**B**) the statuettes.

Figure 5 provides scanning electron microscopy (SEM) images that offer valuable insights into the alloy microstructure of the bronze statuettes. SEM is a powerful tool for examining surface morphology and microstructural features at high magnification, making it particularly useful for the analysis of materials like bronze. (A) Alloy microstructure of the plume, In Fig. 5A, we observe an SEM image showcasing the alloy microstructure of the plume, a specific component of the bronze statuette. This microstructural analysis is essential for understanding the condition and composition of this region. The SEM image reveals the topography and surface features of the plume, offering a close-up view of its structure. Key information that can be gleaned from this image includes the distribution of phases within the alloy, grain boundaries, and any signs of corrosion or degradation. The plume’s alloy microstructure is a critical aspect to consider when assessing the overall condition of the bronze statuette. Changes in this microstructure could indicate the impact of burial, environmental factors, or conservation efforts. (B) Alloy microstructure of the statuettes, Fig. 5B presents an SEM image of the alloy microstructure of the statuettes, focusing on a broader view of the statuette’s surface. This image provides a comprehensive look at the alloy’s composition and structural characteristics, which are essential for understanding the overall condition of the statuettes. Similar to the plume, this image allows for the examination of features like grain boundaries, crystallographic structure, and any indications of corrosion or surface alterations. It’s a critical component in assessing the impact of burial and environmental factors on the statuettes. These SEM images serve as a foundation for detailed material analysis. Researchers can use them to identify changes in the alloy microstructure caused by corrosion, material degradation, or chemical interactions with the burial environment. They also help in evaluating the effectiveness of conservation and restoration efforts. If there are significant alterations or signs of corrosion in the microstructure, conservation strategies can be tailored to address these specific issues. In summary, Fig. 5’s SEM images provide valuable visual data for understanding the alloy microstructure of both the plume and the statuettes. These images are instrumental in the study of how burial and environmental factors influence the microstructural integrity of these bronze artifacts.

#### Alloy chemical composition

**Figure 6:**
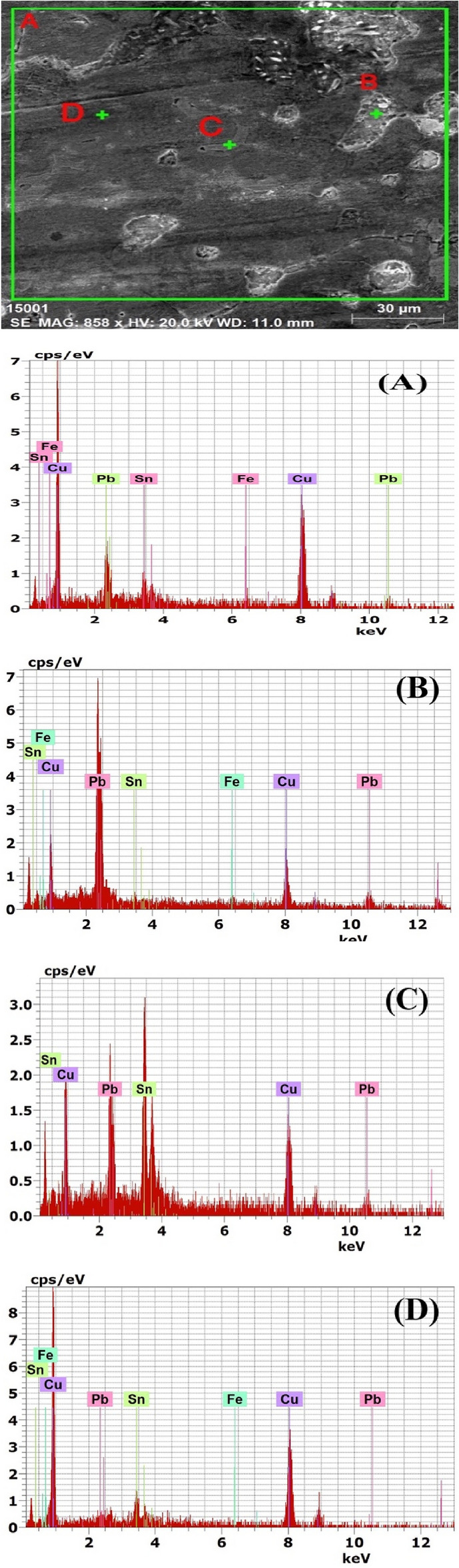
Shows the sites of the SEM image and the corresponding EDS analysis of the statuette alloy.

The chemical composition of the alloy was analyzed using SEM/EDS, and the results are presented in Table 3 and Fig. 6. The alloy is composed of 73.49% copper, 9.96% tin, and 15.36% lead. The corroded surface was also analyzed using non-destructive in situ pXRF, and the results are presented in Table 4. The analysis revealed that the alloy used in manufacturing the statuette contained copper ranging from 71 to 76%, tin ranging from 2.5 to 4%, and lead ranging from 10.5 to 14%. The soil components on the surface of the statuette were found to be high in silicon, magnesium, and calcium, while phosphorus, aluminum, and iron were present in very low amounts. These soil elements are commonly found in the weathering coatings of ancient bronzes buried in the soil^57^. The presence of chloride elements may be attributed to dissolved minerals in the surrounding environment. Elemental mapping for copper, tin, and lead in the metal core of the statuette is presented in Fig. 6, which shows that the lead globules are dispersed throughout the alloy structure. EDS and XRF analysis confirmed that the manufacturing alloy of the statuette is leaded bronze, a type of alloy commonly used for making statues in the late period of Egypt^58^.

**Table 3:** EDS analysis of different spots in the statuettes’ alloy.

Spot	Element wt%			
	Fe	Cu	Sn	Pb
A	1.19	73.49	9.96	15.36
B	1.26	28.19	2.30	68.25
C		34.89	39.83	25.28
D	0.15	85.51	12.03	2.31

**Table 4:** XRF results of different spots in the feather, sun disk, and the statuettes (wt%).

Element	Sun disk	Feather	Statuettes
Mg	1.343	1.547	1.131
Al	0.422	0.28	0.551
Si	4.879	4.828	5.763
P	0.491	0.197	0.241
Cl	0.361	0.474	1.99
Ca	2.346	0.956	1.598
Fe	0.488	0.26	0.314
Ni	0.262	0.316	0.219
Cu	70.869	75.418	74.921
As	1.1821	0.271	0.329
Sn	3.564	4.137	2.384
Pb	13.792	11.162	10.558

Table 3 presents the results of Energy-Dispersive X-ray Spectroscopy (EDS) analysis conducted on different spots in the alloy of the statuettes. EDS is a powerful analytical technique used to determine the elemental composition of a material by measuring the characteristic X-rays emitted from the sample when it is bombarded with electrons. In this context, EDS is employed to gain insights into the composition of the bronze alloy at various locations on the statuettes’ surface. Here, Table 3 provides the weight percentages (wt%) of four key elements. Iron (Fe), Copper (Cu), Tin (Sn), and Lead (Pb). These elements are primary constituents of bronze alloys, and their presence and distribution can reveal crucial information about the alloy’s composition. In spot A: Iron (Fe): 1.19%, Copper (Cu): 73.49%, Tin (Sn): 9.96% and Lead (Pb): 15.36%. In spot B: Iron (Fe): 1.26%, Copper (Cu): 28.19%, Tin (Sn): 2.30% and Lead (Pb): 68.25%. In spot C: Copper (Cu): 34.89%, Tin (Sn): 39.83%, and Lead (Pb): 25.28%. In spot D: Iron (Fe): 0.15%, Copper (Cu): 85.51%, Tin (Sn): 12.03% and Lead (Pb): 2.31%. Table 3 reveals a significant variation in the alloy composition at different spots on the statuettes. Notably, the percentages of copper, tin, and lead vary considerably among the analyzed spots. This heterogeneity can provide insights into the alloy’s manufacturing process, regional differences in composition, or localized corrosion effects. The presence of iron in the alloy, even in relatively small amounts, is indicative of impurities in the bronze. Iron is not a typical component of bronze alloys and can affect their properties, including corrosion resistance. The presence of iron in spots A and D is worth further investigation. Spot B exhibits the highest lead content, comprising 68.25% of the alloy composition. Such a high lead content suggests that this particular spot may have been intentionally alloyed or contaminated with lead. The reasons for this localized high lead content should be explored. Copper and tin are the primary components of bronze alloys. In spots C and D, the copper content is significantly higher than tin, indicating variations in the copper-to-tin ratio. These variations could affect the alloy’s properties and corrosion susceptibility. The variations in alloy composition may correlate with the observed corrosion patterns on the statuettes. Differences in composition can influence the formation of corrosion products and, subsequently, the preservation challenges. In summary, EDS analysis, as presented in Table 3, highlights the heterogeneity and impurity levels within the bronze alloy of the statuettes. These variations in composition could have implications for the corrosion patterns and preservation strategies. Further investigations into the sources of these variations and their impact on the statuettes’ condition are warranted.

Table 4 provides the results of X-ray Fluorescence (XRF) analysis for various elements (expressed in weight percentages, wt%) in different parts of the Feather, Sun Disk, and the Statuettes. XRF is a non-destructive analytical technique used to determine the elemental composition of materials, and in this context, it helps in understanding the chemical composition of these archaeological artifacts. Let’s discuss the significance of these findings. In the sun disk, the Sun Disk exhibits a high copper (Cu) content of 70.869%. This high Cu content is consistent with the bronze material commonly used in ancient artifacts, as Cu is a primary component of bronze. Lead (Pb) is also significantly present at 13.792%, indicating potential lead alloying or contamination, which could affect the artifact’s properties and corrosion patterns. In feather, the Feather, compared to the Sun Disk, shows a lower Cu content at 75.418%. This difference in copper content may suggest variations in the alloys used for different parts of the artifact. The Feather exhibits higher tin (Sn) content at 4.137% compared to the Sun Disk, which is an interesting variation and may influence corrosion behavior. In statuettes, similar to the Feather, the Statuettes show a substantial Cu content at 74.921%, reinforcing that bronze, rich in copper, is the primary material. The Statuettes have a relatively high chlorine (Cl) content at 1.99%, which may be associated with burial conditions or previous conservation efforts involving chloride-containing compounds. Regarding the common elements Magnesium (Mg), aluminum (Al), silicon (Si), and calcium (Ca) are present in relatively similar amounts in all three parts of the artifact, likely indicating shared compositional elements. By comparing feathers and statuettes, the Feather exhibits a slightly higher tin (Sn) content compared to the Statuettes. This difference in Sn content might suggest variations in the alloy compositions used for different parts of the artifact. All parts of the artifact contain a high copper content, consistent with bronze composition. Copper is a key component of bronze and plays a significant role in determining its properties. The presence of lead (Pb), chlorine (Cl), and arsenic (As) may require further investigation, as these elements can impact the corrosion and degradation of artifacts. In summary, the XRF results in Table 4 offer valuable insights into the elemental composition of different parts of the artifact. Variations in the composition, particularly in the content of copper and other elements, may provide clues about the alloying, manufacturing techniques, or environmental conditions to which the artifact has been exposed. These findings are important for understanding the artifact’s corrosion behavior and for making informed decisions regarding its preservation and conservation. Further research is needed to interpret these findings in the context of the artifact’s history and archaeological significance.

#### Corrosion products analysis

**Figure 7:**
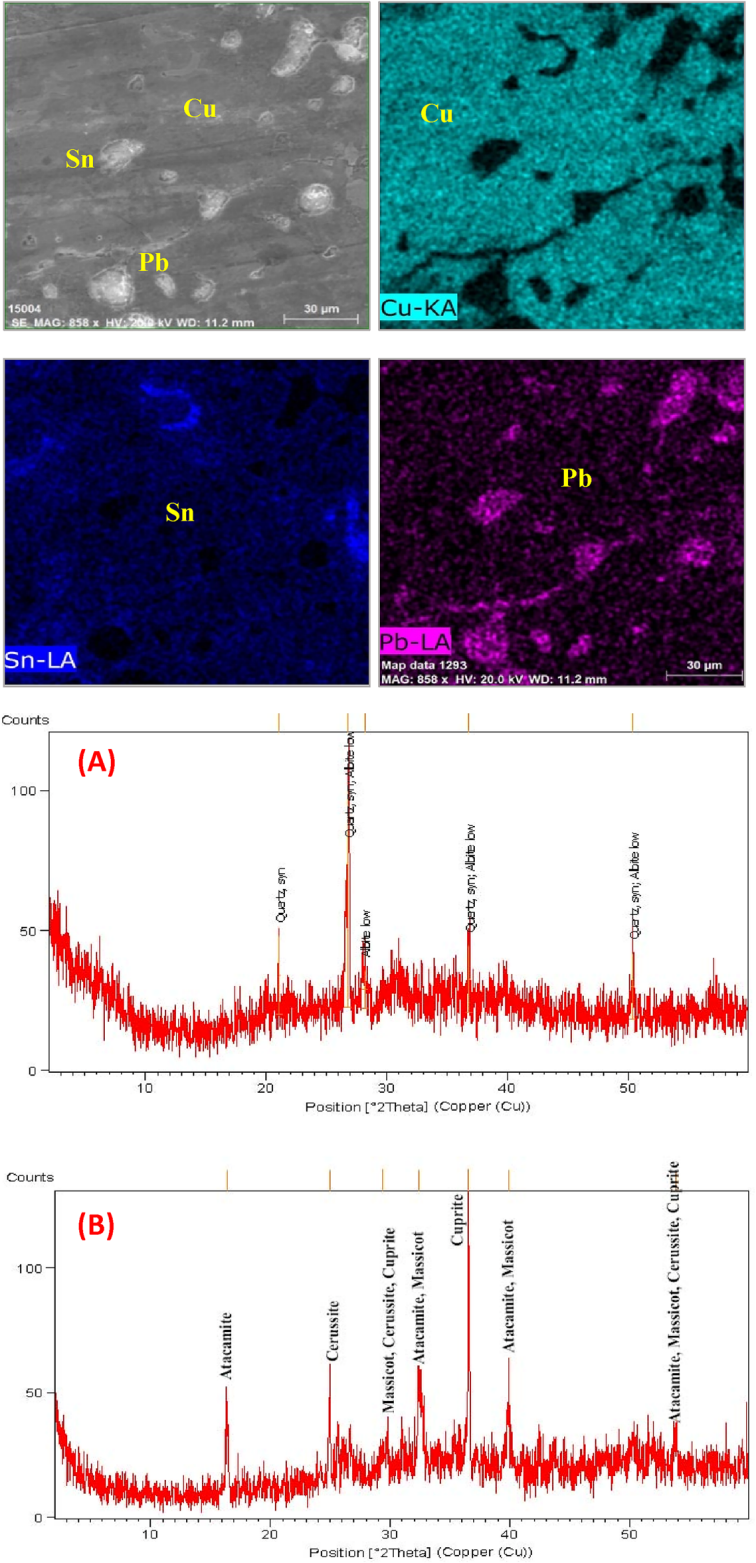
(**A**) XRD results of the Ehnasya soil sample and (**B**) XRD analysis of the corrosion products.

Figure 7b and Table 5 exhibit the principal substances resulting from corrosion that were detected through X-ray diffractometry. Meanwhile, Fig. 7a and Table 6 display the mineral composition of the soil. The XRD analysis revealed that the soil sample examined is composed primarily of quartz (91%), with a small amount of albite (9%). As stated above, *p*XRF analysis of the soil deposits covered the statuettes showing soil components such as Si, Ca, Mg, P, Fe, and Al. The cultivated lands are examined in the Nile valley, and they found that it is a predominated sandy silt soil type^59^. The gained corrosion products of a particular soil are often attributable to several soil properties that interact to make the soil corrosive to buried copper. If the agricultural usage of the land ceases, the addition of lime may also be stopped, reducing the pH level from 6 to 5 in clay soils, or to about 4–4.5 in sandy soils. At the same time, draining wetlands may cause dramatic acidification of soils owing to the amounts of sulfur compounds that would be released and the resulting sulfide oxidations. The main corrosion products below the sandy encrustation layer consist of cuprite (Cu_2_O), atacamite (Cu_2_(OH)_3_CI), Cerussite PbCO_3,_ and Massicot PbO, as presented in Fig. 7b. The existence of significant quantities of copper oxychlorides in the deterioration results would be expected for bronze statuettes that had been buried in soil with a high chloride content (Megahed et al., 2022). In environments rich in chloride, cuprous chloride (known as nantokite or CuCl) can form at the interface between the external patina and the metal that still exists. When nantokite is exposed to oxygen and water in the environment, it turns into atacamite and other phases, which then react with copper to create new cuprous chloride and water. This is a continuous process where copper, chlorine, oxygen, and water transform into cuprite and atacamite. The presence of copper oxychlorides is particularly hazardous in corrosion as it makes the metal surface unstable. The deterioration of the statuettes showed high levels of lead content, which were indicated by the presence of lead (II) oxide (massicot or PbO) and carbonate (cerussite or PbCO_3_)^60^.

**Table 5:** XRD results of Ehnasya soil sample.

Ref. code	Mineral name	Chemical formula	Semi quant (%)
01-082-0511	Quartz	SiO_2_	91
01-074-6368	Albite	Na(AlSi_3_O_8_)	9

**Table 6:** XRD results of the corrosion products.

Ref. code	Mineral name	Chemical formula	Semi quant (%)
05-0667	Cuprite	Cu_2_O	40.24
02-0146	Atacamite	Cu_2_Cl(OH)_3_	32.19
05-0421	Cerussite	PbCO_3_	18.14
05-0570	Massicot	PbO	9.43

Table 5 presents the X-ray Diffraction (XRD) results of a soil sample from Ehnasya, providing information about the minerals found within the sample. XRD is a powerful analytical technique used to identify the mineralogical composition of geological materials. In this specific soil sample, two minerals have been identified, and their respective semi-quantitative percentages are given. Let’s discuss the significance of these findings. Quartz (SiO2), quartz is the dominant mineral in the Ehnasya soil sample, constituting a significant semi-quantitative percentage of 91%. Quartz is one of the most common minerals in the Earth’s crust and is known for its high resistance to weathering and erosion. Its presence in the soil indicates a source of silica and suggests that the soil may be derived from rocks rich in quartz. Albite (Na(AlSi_3_O_8_)), albite is also identified in the soil sample, comprising a semi-quantitative percentage of 9%. Albite is a plagioclase feldspar, a group of rock-forming minerals commonly found in igneous and metamorphic rocks^61^. Its presence in the soil suggests that the soil may have originated from rocks containing plagioclase minerals. The XRD results for this soil sample provide insights into its mineralogical composition. The dominance of quartz, a highly stable mineral, suggests that the soil may have undergone minimal alteration or weathering processes. The presence of albite indicates the potential influence of rocks rich in feldspar minerals in the soil’s formation. These findings are significant for various reasons. Understanding the mineralogical composition of the soil is crucial for geological and environmental studies. It can provide insights into the soil’s origin, geologic history, and potential sources of sediment. The presence of specific minerals can impact the soil’s properties, including its ability to retain water, nutrient content, and erosion resistance. In the context of archaeological or conservation research, knowledge of the soil composition around buried artifacts can be essential. It can affect the corrosion behavior and preservation of artifacts, as different minerals can have varying effects on the surrounding environment. In summary, the XRD results presented in Table 5 contribute to a better understanding of the mineralogical composition of the Ehnasya soil sample, which has implications for both geological studies and potential implications for archaeological and conservation efforts in the region.

Table 6 provides X-ray Diffraction (XRD) results for the corrosion products found on the examined bronze statuettes. Table 6 lists the identified minerals, their chemical formulas, and the semi-quantitative percentages of each mineral. These results are crucial for understanding the nature of the corrosion and the chemical reactions taking place on the statuettes. Let’s discuss the significance of these findings. Cuprite (Cu_2_O), cuprite is a significant component of the corrosion products, constituting a semi-quantitative percentage of 40.24%. Cuprite is a copper oxide mineral that often forms as a corrosion product on copper and copper alloy artifacts^62^. It is typically reddish-brown and is a common product of copper corrosion in the presence of oxygen and moisture. Atacamite (Cu_2_Cl(OH)_3_), atacamite is another copper-containing mineral identified, making up a substantial portion with a semi-quantitative percentage of 32.19%. Atacamite is a chlorine-rich copper mineral often found in association with cuprite on corroded copper surfaces. Its presence indicates the influence of chloride ions on the corrosion process. Cerussite (PbCO_3_), cerussite, a lead carbonate mineral, is also identified as part of the corrosion products, with a semi-quantitative percentage of 18.14%. Cerussite can form a corrosion product on lead-based artifacts and is often associated with the presence of carbon dioxide and carbonate ions. Massicot (PbO), massicot, a lead oxide mineral, is present in the corrosion products, although to a lesser extent, with a semi-quantitative percentage of 9.43%. Massicot typically forms as a result of the oxidation of lead and is one of the oxidation products of lead corrosion. The XRD results in Table 6 reveal the composition of the corrosion products on the bronze statuettes. These minerals are typical corrosion products associated with copper and lead-based alloys. Their presence provides important insights into the corrosion processes that have occurred^63^. The presence of cuprite and atacamite suggests that the corrosion of the bronze statuettes is predominantly copper-based. Cuprite is often formed when copper reacts with oxygen, while atacamite can result from the interaction of copper, chloride ions, and water. The identification of cerussite and massicot indicates lead-based corrosion products. Lead corrosion often results in the formation of lead oxide and carbonate compounds. Understanding the specific corrosion products on the statuettes is valuable for several reasons. Knowing the composition of the corrosion products is essential for developing effective conservation strategies to stabilize and protect the artifacts. The presence of these minerals can provide insights into the environmental conditions and burial history of the statuettes. These findings contribute to the broader field of materials science and corrosion science, aiding in the understanding of metal degradation processes. In summary, the XRD results in Table 6 shed light on the composition of the corrosion products on the bronze statuettes. The identification of cuprite, atacamite, cerussite, and massicot helps researchers and conservators make informed decisions about the preservation and restoration of these valuable cultural artifacts^64^.

### Electrochemical behavior of bronze in the soil

Bronze is an alloy composed of copper and tin, with the addition of other elements such as zinc, lead, and nickel. It has been widely used for various applications, including art, architecture, and coins, due to its unique properties such as durability, ductility, and corrosion resistance. However, when exposed to soil, bronze can undergo various electrochemical reactions that affect its behavior and integrity. In soil, bronze is subjected to various electrochemical reactions due to the presence of different ions, such as chloride, sulfate, and bicarbonate^65^. These ions can interact with the bronze surface and cause electrochemical corrosion, leading to the formation of a patina layer on the surface. The patina layer is a complex mixture of copper compounds, such as cuprite, tenorite, malachite, and azurite, and provides a protective barrier that prevents further corrosion. Another electrochemical reaction that can occur with bronze in the soil is galvanic corrosion. Galvanic corrosion occurs when two dissimilar metals are in contact in the presence of an electrolyte, and a potential difference is created, leading to the transfer of electrons from the anode to the cathode. This reaction can occur with bronze and other metals, such as iron or steel, resulting in the corrosion of the more active metal. Furthermore, soil composition, moisture content, and pH can affect the electrochemical behavior of bronze. For example, acidic soils can increase the rate of corrosion of bronze, while alkaline soils can cause bronze to form a protective layer more rapidly. In conclusion, the electrochemical behavior of bronze in the soil is complex and depends on various factors such as soil composition, moisture content, and pH. While bronze is generally resistant to corrosion, it can undergo various electrochemical reactions that can affect its integrity and durability in soil^66^.

#### Weight loss measurements

**Figure 8:**
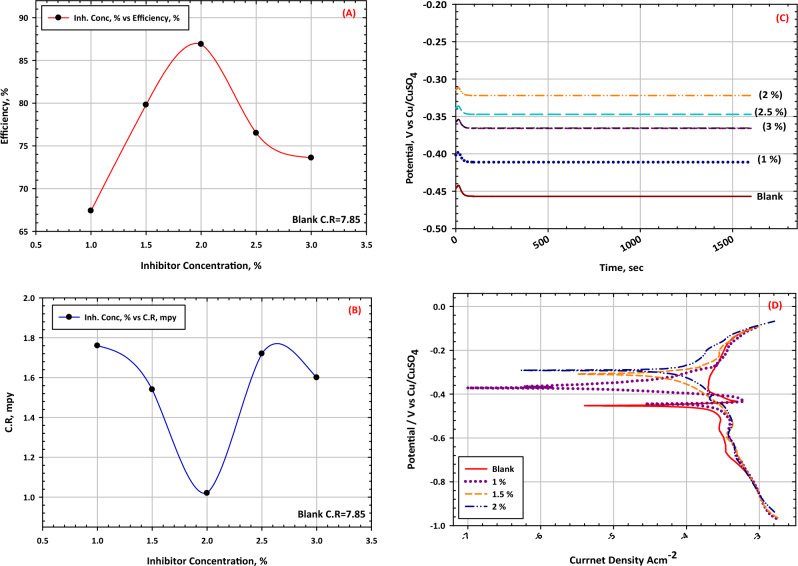
Inhibition efficiency and average corrosion rate of the bronze alloy during exposure in the soil for different concentrations of inhibitor mixture (benzotriazole BTA 3% in alcohol, then final protection was carried out using two layers of paraloid PA 3% in alcohol) (**A**) inhibition efficiency and (**B**) average corrosion rate. Electrochemical measurements of bronze alloy different concentration from conservation mixture in corrosive soil (**C**) open‐circuit potential, (**D**) Potentiodynamic polarization curves.

The relationship between weight loss measurements and corrosion of leaded bronze in the soil is like that in marine environments. In soil, corrosion of leaded bronze can occur due to factors such as the moisture content, pH level, and chemical composition of the soil. Weight loss measurements can be used to assess the degree of corrosion in leaded bronze in soil by measuring the difference in weight of the material before and after exposure to the soil environment. In soil environments, weight loss measurements can be used to monitor the corrosion of leaded bronze, and to determine the appropriate maintenance and protection measures necessary to prevent further corrosion. For example, if weight loss measurements indicate that the leaded bronze is corroding at a faster rate than expected, it may be an indication that the soil environment is more corrosive than anticipated, or that the corrosion protection measures in place are not adequate^67^. In some cases, leaded bronze may be buried in soil as a part of the infrastructure, such as pipelines or buried structures. The exposure of leaded bronze to soil can cause localized corrosion, and weight loss measurements can be a useful tool to assess the degree of corrosion and determine appropriate maintenance measures see Fig. 8a,b. In addition, weight loss measurements can be used to compare the corrosion resistance of different materials in soil environments. Overall, weight loss measurements can be used to assess the degree of corrosion of leaded bronze in soil environments and to guide maintenance and protection efforts to prevent further corrosion. These measurements can help to ensure the longevity of leaded bronze infrastructure buried in soil and to help prevent environmental damage and potential safety hazards that can result from corroded infrastructure. Figure 8a represents an increasing tendency for weight reduction but Fig. 8b, represents the lower rate of corrosion of leaded bronze after being buried in soil for a certain period and these results are matched with the obtained equation from Fig. 8b.

#### Open circuit potential and polarization curves

Figure 8c shows the open circuit potential curves of leaded bronze after exposure to different levels of an inhibitive mixture. The corrosion potential shifts towards the positive direction until reaching a concentration of 2%. After this concentration, increasing the mixture concentration causes the corrosion potential to shift back toward the negative direction. This indicates that the optimal concentration for the coating mixture is 2%. In Fig. 8d, the potentiodynamic polarization curves of leaded bronze after exposure to different concentrations of the inhibitive mixture are displayed. The current density and Tafel slope were calculated from fitting curves. There were some differences observed, such as a positive shift in the corrosion potentials and a decrease in the current density with increasing levels of the inhibitive mixture. The corrosion current density can be used to evaluate the corrosion rate, which suggests that the corrosion rates decrease with increasing amounts of the inhibitive mixture. On the anodic branch, bronze exposed to different concentrations showed significant disintegration, while linear Tafel areas were observed on both the anodic and cathodic branches^68^.

#### Electrochemical impendence spectroscopy

**Figure 9:**
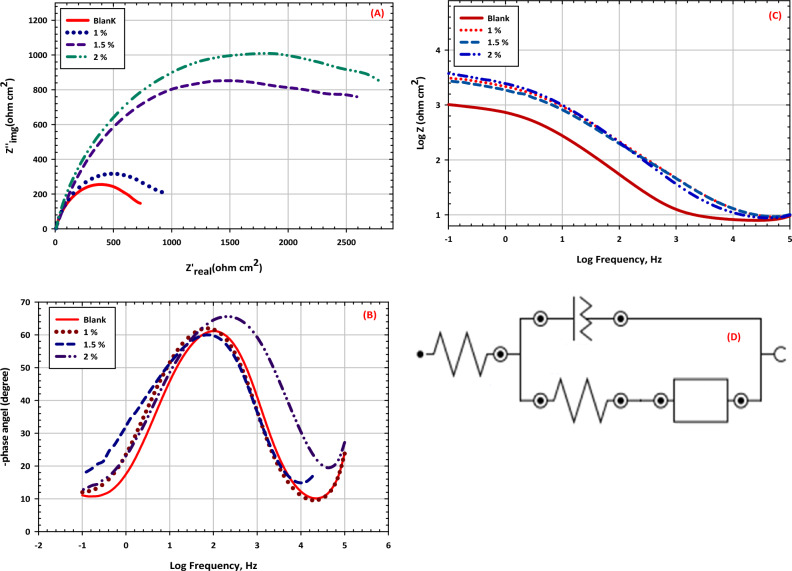
EIS curves of bronze alloy different concentration from conservation mixture in corrosive soil, (**A**) Plots of Nyquist diagrams, (**B**) Plots of Bode plots, (**C**) Plots of phase fitted results, (**D**) The obtained equivalent circuit by fitting the results.

Electrochemical impedance spectroscopy (EIS) is a powerful technique for characterizing the electrochemical behavior of materials. When applied to leaded bronze in soil, EIS can provide valuable insights into the corrosion behavior of this material in its environment. Leaded bronze is an alloy of copper, tin, and lead that is commonly used in applications such as statues, monuments, and building facades. When exposed to soil, leaded bronze can undergo corrosion, which can lead to aesthetic and structural damage. EIS can be used to study this corrosion process by measuring the electrical impedance of the material as a function of frequency. In EIS, a small amplitude alternating current is applied to the material, and the resulting voltage response is measured^69^. The impedance of the material is then calculated as the ratio of the voltage response to the applied current. By varying the frequency of the applied current, EIS can provide information about the capacitive and resistive components of the material’s impedance. When applied to leaded bronze in soil, EIS can provide information about the formation and growth of corrosion products on the surface of the material. For example, as the material corrodes, a layer of copper oxide may form on the surface. This layer can act as a barrier to further corrosion, but it can also increase the capacitive component of the material’s impedance. By analyzing the impedance data as a function of frequency, researchers can gain insights into the formation and growth of this oxide layer, as well as the overall corrosion behavior of the material in the soil^70^. Overall, EIS is a valuable tool for studying the electrochemical behavior of leaded bronze in soil. By providing information about the impedance of the material as a function of frequency, EIS can provide insights into the corrosion behavior of the material and can be used to develop strategies for mitigating this corrosion. In Fig. 9, EIS plots of leaded bronze in different exposure periods during an immersion test in soil with 20 wt% water content is compared at open circuit potential (OCP). The EIS plots show similar features, with a low-frequency loop indicating a semi-infinite diffusion process and a high-frequency loop with a high-phase shift induced by Cu/CuSO_4_ and/or the potentiostat in low soil conductivity. The high phase shift is commonly observed in soil corrosion systems and other porous media systems, such as bronze and aged bronze covered with corrosion products. The equivalent electrical circuit in Fig. 9D can be used to characterize the corrosion system^71^. In electrochemical impedance spectroscopy (EIS), an equivalent circuit is used to model the electrochemical processes occurring at the electrode–electrolyte interface. The elements in this circuit represent different physical phenomena. Here’s an explanation of the common electrical elements that might be proposed in an equivalent circuit, such as the one depicted in Fig. 9D. R_s_ represents the resistance of the electrolyte solution between the working electrode and the reference electrode. It accounts for the ohmic drop in the solution and is usually depicted as a resistor in the equivalent circuit. R_ct_ represents the resistance to the transfer of electrons across the electrode/electrolyte interface^72^. A higher Rct indicates a slower rate of electrochemical reactions, which often corresponds to a less corrosive environment or more protective corrosion products. C_dl_ presents the capacitance of the electrical double layer formed at the interface between the electrode surface and the electrolyte. It accounts for the storage of charge in the double layer and is influenced by factors such as electrode surface area and electrolyte composition. The Q or CPE is used to represent non-ideal capacitive behavior, often due to surface roughness, inhomogeneities, or porosity of the electrode. Instead of a perfect capacitor, the CPE accounts for a more complex, frequency-dependent impedance. In the context of Fig. 9D, R_s_ (Solution Resistance) could be represented by the initial resistor at the beginning of the circuit. R_ct_ (Charge Transfer Resistance) and C_dl_ (Double Layer Capacitance) are often in parallel to represent the charge transfer process at the interface and the capacitance of the double layer. Q (Constant Phase Element) might be used instead of a pure capacitor to model the non-ideal capacitive behavior. The combination of these elements helps to simulate the impedance response of the system and interpret the underlying electrochemical processes influencing the corrosion behavior of the statues.

Table 7 presents the fitting results for leaded bronze after exposure to red soil with different loads of inhibitive mixture, using the reciprocals of Rct to represent the corrosion rate. The corrosion rate of leaded bronze decreases with inhibitive mixture loads, indicating that the formation of an oxide layer could inhibit corrosion, consistent with the results in Fig. 7b. The Rf value of the leaded bronze in the soil increases with exposure time, indicating that new corrosion products are generated and absorbed on the sample surface, enhancing the defensive impact of the oxide layer on bronze^73^.

**Table 7:** Electrochemical parameters and inhibition efficiency for bronze in soil containing different concentrations of inhibitive mixture.

Inhibitive mixture Conc., (%)	*OCP* (V) versus CCE	*E*_*corr*_ (V) veruss CCE	*b*_*a*_ (V dec^−1^)	*b*_*c*_ (V dec^−1^)	*I*_*corr*_ (A cm^−2^)	*C.R.*, mpy	*I.E*, (%)	*R*_*P*_ (Ohm cm^2^)
Blank	− 0.454	− 0.6340	0.8190	− 1.8086	7.5376e−4	5. 580	–	439
1	− 0.372	− 0.5213	0.5620	− 0.4396	2.2460e−4	2. 680	51.9	856
1.5	− 0.305	− 0.3981	0.3592	− 0.3299	7.2e−5	0.798	85.6	870
2	− 0.287	− 0.2192	0.2185	− 0.2418	3.4881e−5	0.566	89.8	982

Table 7 presents a comprehensive overview of the electrochemical parameters and inhibition efficiency for bronze corrosion in soil containing varying concentrations of an inhibitive mixture. This data is essential for evaluating the performance of the inhibitor mixture and understanding how its concentration affects corrosion behavior. Let’s discuss the significance of the findings. Inhibitive Mixture Concentrations (Conc., %), Table 7 includes data for different concentrations of the inhibitive mixture, ranging from 1 to 2%, along with a blank (no inhibitor) for reference. Open-Circuit Potential (OCP) vs. CCE, OCP provides insights into the thermodynamic aspects of the corrosion process. More positive (less negative) OCP values indicate a thermodynamically more stable state. The OCP becomes less negative (i.e., more positive) with increasing inhibitor mixture concentration. This shift suggests that the inhibitive mixture has a positive effect on the corrosion potential, making the system more corrosion resistant. Corrosion potential (Ecorr) vs. CCE; E_corr_ represents the potential at which the corrosion rate is at its lowest^74^. A less negative value suggests a more noble state, indicating lower corrosion activity. Ecorr values become less negative with increasing inhibitor mixture concentration. This shift indicates a reduction in corrosion activity, further supporting the inhibitory effect. Anodic (b_a_) and Cathodic (b_c_) Tafel Slopes (V dec^−1^), Tafel slopes provide information about the reaction kinetics. Lower values suggest faster electrode reactions. The anodic and cathodic Tafel slopes decrease with increasing inhibitor mixture concentration, indicating that the inhibitive mixture facilitates both anodic and cathodic reactions. Corrosion Current Density (Icorr) (A cm^−2^), Icorr represents the rate of corrosion. Lower values indicate reduced corrosion rates. Icorr decreases significantly with increasing inhibitor concentration, highlighting the inhibitor’s effectiveness in reducing the corrosion rate. Corrosion Rate (C.R.) (mpy—mils per year), C.R. is a practical measure of how quickly the material is corroding^75^. Lower values indicate slower corrosion. The corrosion rate drops substantially with higher inhibitor mixture concentrations, demonstrating the inhibitor’s capacity to protect the bronze from corrosion. Inhibition Efficiency (I.E.) (%), I.E. provides a percentage measure of the inhibition’s effectiveness, with higher percentages indicating better protection. The inhibition efficiency increases as the inhibitor mixture concentration rises, with the highest concentration achieving the greatest inhibition. Polarization resistance (R_P_) (Ohm cm^2^), polarization resistance is related to the ease of charge transfer at the metal-electrolyte interface. Higher R_P_ values signify increased resistance to corrosion. R_P_ increases as the inhibitor concentration goes up, suggesting a decrease in the rate of charge transfer and enhanced corrosion resistance^76^. The data in Table 7 clearly demonstrate the inhibitory effect of the inhibitive mixture on bronze corrosion in soil. As the inhibitor mixture concentration increases, various electrochemical parameters, including OCP, Ecorr, Tafel slopes, Icorr, corrosion rate, inhibition efficiency, and polarization resistance, all exhibit trends that indicate improved corrosion protection. Understanding how the inhibitive mixture’s concentration affects these electrochemical parameters is essential for optimizing corrosion protection strategies for bronze artifacts buried in the soil. The data provides valuable insights for conservators and researchers working on the preservation of archaeological and cultural heritage objects. In summary, the results presented in Table 7 underscore the effectiveness of the inhibitive mixture in mitigating bronze corrosion, with higher concentrations of the inhibitor mixture leading to improved corrosion protection and inhibition efficiency^77^.

### Characterization of surface morphology

**Figure 10:**
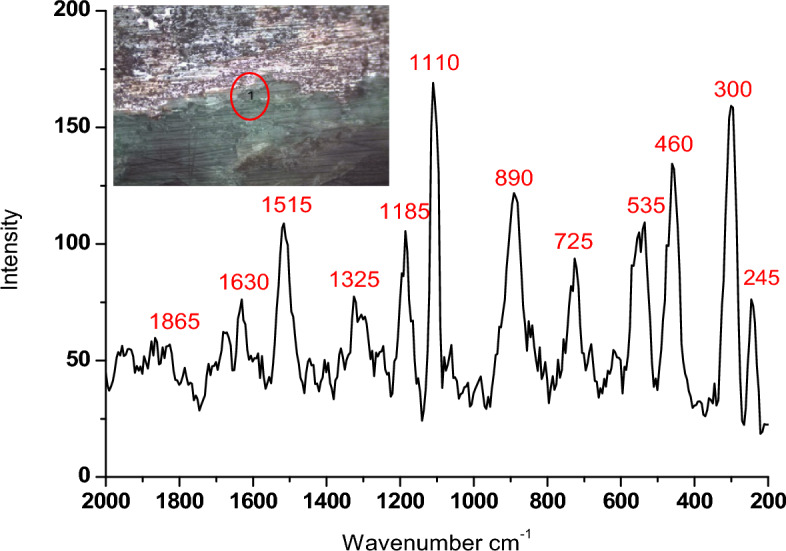
Raman spectral analysis of spot No. 1 in the green patina.

Leaded bronze is an alloy composed of copper, tin, and lead. When leaded bronze is buried in soil, it can undergo various physical and chemical changes that affect its surface morphology. The characterization of surface morphology can provide insight into the degree of degradation and corrosion that has occurred. One method of characterizing surface morphology is through visual inspection and microscopy^78^. By examining the surface of the leaded bronze under a microscope, it is possible to identify changes in texture, color, and shape. Scanning electron microscopy (SEM) can provide high-resolution images that show the details of surface features and any corrosion products that may have formed. Another method is X-ray diffraction (XRD) analysis. This technique can be used to identify the mineral phases present on the surface of the leaded bronze. XRD can also reveal the degree of oxidation or corrosion that has occurred. Additionally, atomic force microscopy (AFM) can be used to measure the roughness of the surface of the leaded bronze. This method can provide detailed information on surface topography and roughness, which can be used to track changes in surface morphology over time^79^. Overall, the characterization of surface morphology can help to understand the mechanisms of degradation and corrosion of leaded bronze in soil environments and guide the development of preservation strategies to mitigate these effects. Figure 10 displays the physical characteristics of how weathering affects bronze surfaces^80^. The pictures demonstrate the noticeable changes in surface morphology from an untreated surface to a surface that has undergone corrosion, as seen in Fig. 10a. Upon exposing the surface to an inhibitive mixture, a protective layer covers the surface and prevents any signs of corrosion, as mentioned in Fig. 10b. As the treated sample is exposed to topsoil for a specific duration, the surface layers gradually bond to the metal surface, and there are no cracks or pits visible on the surface, as shown in Fig. 10b. Observing the corrosion morphology shows the variation in the corrosion process in soil, from non-uniform corrosion to pitting, and then to chloride and sulfide attack. The number and depth of pits increase over time, with the pits expanding laterally and the corrosion process dominated by the creation of new pits and pit development in the depth direction^81^.

### Micro-Raman spectroscopy of the green *patina*

**Figure 11:**
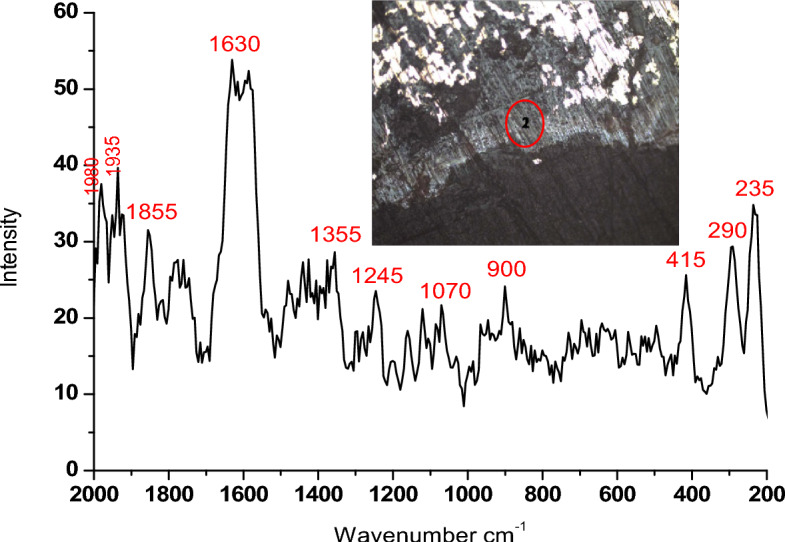
Raman spectral analysis of spot No. 2 in the green patina.

**Figure 12:**
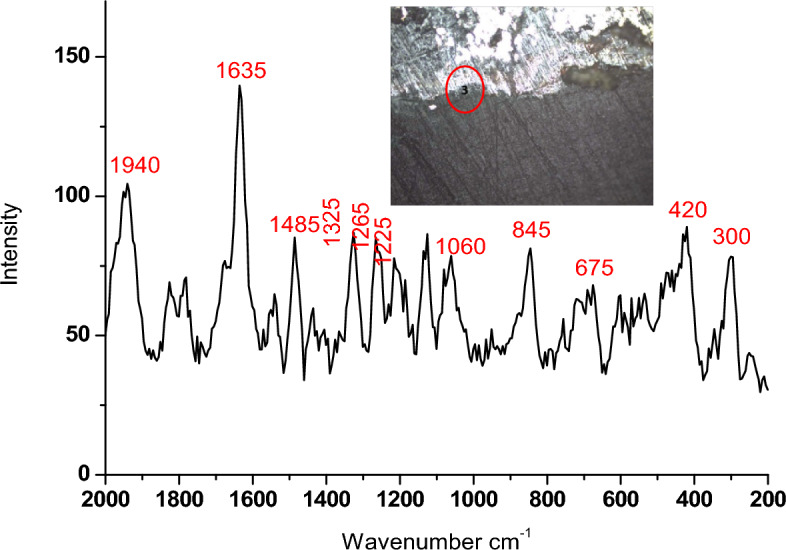
Raman spectral analysis of spot No. 3 in the green patina.

Figures 10, 11 and 12 display the Raman spectra of various locations within the patina cross-section, revealing corrosion products that are identified based on the Raman bands specified in Tables 6, 7 and 8. The Raman spectrum analysis in Fig. 10 indicated the presence of brochantite (Cu_4_(SO_4_)(OH)_6_) through three distinct bands; a highly prominent band at 1110 cm^−1^, a strong peak at 300 cm^−1^, and a moderate peak at 245 cm^−1^^82–84^. Additionally, Raman spectroscopy detected the existence of two chloride phases in the green patina, comprising botallackite and paratacamite. Botallackite (Cu_2_(OH)_3_Cl) is characterized by distinctive bands at 460 and 890 cm^−1^^85,86^ and Paratacamite (Cu_2_(OH)_3_Cl) at 725, 890 cm^−1^. Figures 11 and 12 presented two distinct sulfate compounds observed in the green patina. The findings indicated the presence of brochantite (Cu_4_(SO_4_)(OH)_6_) with prominent bands identified at (235, 290, 300, 420 cm^−1^) and several moderate bands at (415, 1070, 1265 cm^−1^). Additionally, antlerite (Cu_3_(SO_4_)(OH)_4_) was detected with characteristic bands at (290, 900, 420, and 845 cm^−1^)^87^. Brochantite is rarely documented in buried archaeological bronzes^88^. The presence of both brochantite and antlerite could be linked to sulfur pollutants formed due to the decomposition of organic matter by sulfate-reducing bacteria in the soil^89^.

**Table 8:** Raman spectral analysis of spot No. 1.

Name	Composition	Detected Raman bands (cm^−1^)	References
Brochantite	Cu_4_(SO_4_)(OH)_6_	245, 300, 1110	^93–95^
Botallackite	Cu_2_(OH)_3_Cl	460, 890	^91,92^
Paratacamite	Cu_2_(OH)_3_Cl	725, 890	^93–95^

Table 8 presents the results of Raman spectral analysis conducted on spot No. 1, showcasing the composition, and detected Raman bands along with relevant references. This analysis is crucial for identifying the specific corrosion products and minerals present, shedding light on the corrosion processes and environmental conditions. Let’s delve into the discussion of these findings. Brochantite (Cu_4_(SO_4_)(OH)_6_), Raman bands detected at 245, 300, and 1110 cm^−1^ are consistent with the presence of brochantite. These Raman bands agree with previous studies, confirming the identification of brochantite in spot No. 1. Brochantite is a copper sulfate hydroxide mineral commonly formed in the corrosion process of copper-based materials. Botallackite (Cu_2_(OH)_3_Cl), Raman bands detected at 460 and 890 cm^−1^ indicate the presence of botallackite^90^. These findings align with previous research, supporting the identification of botallackite in spot No. 1. Botallackite is a copper hydroxy chloride mineral formed as a corrosion product when copper is exposed to chloride-containing environments. Paratacamite (Cu_2_(OH)_3_Cl), Raman bands detected at 725 and 890 cm^−1^ suggest the existence of paratacamite. These findings are consistent with previous studies, confirming the presence of paratacamite in spot No. 1^91^. Paratacamite is another copper hydroxy chloride mineral formed in chloride-rich corrosion environments. The Raman spectral analysis of spot No. 1 reveals the coexistence of brochantite, botallackite, and paratacamite, all of which are corrosion products commonly associated with copper and bronze artifacts exposed to various environmental conditions. The detection of these minerals provides valuable information about the corrosion history of the bronze statuettes and the specific environmental factors at play. The identification of these minerals contributes to a deeper understanding of the corrosion mechanisms affecting the bronze statuettes^92^. This knowledge is essential for conservation efforts, as it guides the development of appropriate strategies for preserving and protecting these valuable artifacts. Additionally, it aids in assessing the environmental conditions at the burial sites, which can inform future archaeological investigations. In summary, the Raman spectral analysis in Table 8 offers valuable insights into the corrosion products present in spot No. 1 of the bronze statuettes, highlighting the complex interplay between the artifacts and their burial environment.

Table 9 presents the results of the Raman spectral analysis conducted on spot No. 2, providing insights into the composition of the corrosion products and minerals detected in this area. Here is the discussion of the findings. Brochantite (Cu_4_(SO_4_)(OH)_6_), Raman bands detected at 235, 290, 415, and 1070 cm^−1^ are indicative of the presence of brochantite. These Raman bands align with the characteristics associated with brochantite, as reported in prior studies^96–98^. Brochantite is a copper sulfate hydroxide mineral often formed as a corrosion product when copper-based materials are exposed to sulfide-rich environments. Antlerite (Cu_3_(SO_4_)(OH)_4_), Raman bands detected at 290 and 900 cm^−1^ suggest the existence of antlerite. These findings are consistent with the Raman bands reported for antlerite in previous research^99–102^. Antlerite is another copper sulfate hydroxide mineral that can form as a corrosion product in the presence of sulfur compounds. The Raman spectral analysis of spot No. 2 confirms the presence of both brochantite and antlerite. These minerals are commonly associated with copper and bronze corrosion products and are formed in specific environmental conditions, particularly those influenced by sulfur compounds^103^. The identification of brochantite and antlerite in spot No. 2 contributes to a more comprehensive understanding of the corrosion history of the bronze statuettes. It indicates that these areas were likely exposed to sulfur-containing compounds, which influenced the formation of these specific corrosion products. This knowledge is essential for conservation efforts and helps guide strategies for preserving the artifacts effectively. In summary, Table 9’s Raman spectral analysis provides valuable insights into the composition of corrosion products in spot No. 2 of the bronze statuettes. The presence of brochantite and antlerite underscores the complexity of the corrosion processes and highlights the significance of environmental factors in the degradation of these artifacts^104^.

**Table 9:** Raman spectral analysis of spot No. 2.

Name	Composition	Detected Raman bands (cm^−1^)	References
Brochantite	Cu_4_(SO_4_)(OH)_6_	235, 290, 415, 1070	^105, 106^
Antlerite	Cu_3_(SO_4_)(OH)_4_	290, 900	^105, 106^

Table 10 provides the results of Raman spectral analysis for spot No. 3, shedding light on the composition of corrosion products and minerals detected in this specific location. Here is the discussion of the findings. Brochantite (Cu_4_(SO_4_)(OH)_6_), Raman bands detected at 300, 420, and 1265 cm^−1^ are indicative of the presence of brochantite. These Raman bands correspond with the characteristic vibrations associated with brochantite, consistent with previous research^105,106^. Brochantite is a copper sulfate hydroxide mineral commonly formed as a corrosion product when copper-based materials are exposed to sulfide-rich environments. Antlerite (Cu_3_(SO_4_)(OH)_4_), Raman bands detected at 420 and 845 cm^−1^ suggest the existence of antlerite. These findings align with the Raman bands associated with antlerite, as reported in previous studies^107^. Antlerite is another copper sulfate hydroxide mineral that forms as a corrosion product, especially in the presence of sulfur compounds. The Raman spectral analysis of spot No. 3 confirms the presence of brochantite and antlerite. These minerals are typical corrosion products associated with copper and bronze artifacts, and their presence suggests exposure to sulfur compounds or sulfide-rich environments, contributing to their formation^108^. The identification of brochantite and antlerite in spot No. 3 provides insights into the corrosion history of the bronze statuettes. It indicates the role of environmental conditions, particularly those influenced by sulfur-containing compounds, in the formation of these specific corrosion products. This information is valuable for developing conservation strategies tailored to the artifacts’ unique corrosion patterns. In summary, Table 10’s Raman spectral analysis enhances our understanding of the composition of corrosion products in spot No. 3 of the bronze statuettes. The detection of brochantite and antlerite underscores the impact of environmental factors on the degradation of these artifacts and informs conservation efforts.

**Table 10:** Raman spectral analysis of spot No. 3.

Name	Composition	Detected Raman bands (cm^−1^)	References
Brochantite	Cu_4_(SO_4_)(OH)_6_	300, 420, 1265	^67,69^
Antlerite	Cu_3_(SO_4_)(OH)_4_	420, 845	^68^

### Conservation treatment

**Figure 13:**
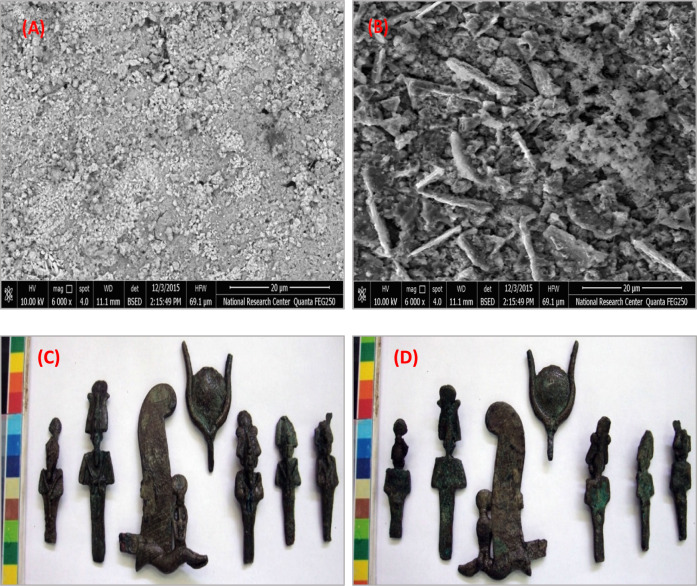
SEM Microscopic morphologies of bronze samples, (**A**) Before treatment, (**B**) After treatment and the statuettes after treatment processes, (**C**) Front side, (**D**) Back side.

Leaded bronze artifacts buried in soil can undergo corrosion and degradation over time, making conservation techniques essential for their preservation. Here are some different techniques for conserving buried leaded bronze in soil. The first step in conserving buried leaded bronze is to excavate the artifact carefully and remove any soil and debris that may be present on its surface^109^. This can be done using hand tools, brushes, and water. Chemical treatments can be used to remove corrosion products and stabilize the metal. For example, tannic acid can be used to remove surface corrosion, while ethylenediaminetetraacetic acid (EDTA) can be used to remove deeper corrosion products. Applying a coating to the surface of the bronze can help to protect it from further corrosion. One option is microcrystalline wax, which can be applied using a brush or sprayer. Electrochemical methods can be used to remove corrosion products and reduce the amount of corrosion occurring on the surface of the bronze^110^. For example, electrolysis can be used to remove corrosion products while also depositing a protective layer of metal ions onto the surface of the bronze. Finally, controlling the environment in which the artifact is stored or displayed can help to prevent further corrosion. This can involve controlling temperature, humidity, and exposure to light, among other factors. Figure 13c, d show the statuettes after undergoing conservation and treatment procedures. To remove soil deposits and powdery corrosion, brushes, needles, and scalpels were used during the cleaning process. A 5% citric acid solution was then applied to eliminate corrosion products, including Cu^+^ and Cu^+2^ oxidization results such as Cu_2_(OH)_3_Cl, Cu_2_(OH)_2_CO_3_, CuO, and Cu_2_O. In addition, citric acid can form stable complexes with Cu (II) compounds, according to MacLeod (1987)^111^. The objects were then rinsed with flowing water followed by distilled water to remove any excess acid and then dried in an oven at 50 °C. After cleaning and rinsing, the statuettes were treated with a 2% benzotriazole solution in ethanol. This solution acts as a physical barrier between CuCl and moisture from the atmosphere, which could activate the CuCl and cause bronze disease, according to Sease (1976)^112^. Finally, two layers of 2% paraloid in ethanol were applied to provide further protection. The objects were then stored in sealed polyethylene food storage boxes with silica gel to eliminate humidity from the air inside the storage container, following MacLeod’s (1987) instructions^89^.

## Conclusion

Corrosion is an inevitable process affecting metals when exposed to elements such as oxygen and moisture. Leaded bronze artifacts buried underground are particularly vulnerable due to their interaction with soil and groundwater, which often contain acids, salts, and other corrosive agents. This corrosion can degrade metal artifacts over time, resulting in material loss and structural damage. Preserving buried leaded bronze artifacts is crucial to maintaining their historical, cultural, and scientific significance. The conservation process aims to prevent or slow down corrosion and stabilize the artifact’s condition. Various techniques are employed, including mechanical cleaning, chemical cleaning, and protective coatings. Mechanical cleaning uses tools like brushes and scalpels to remove surface debris and corrosion products, but it can damage fragile artifacts. Chemical cleaning involves chemical solutions to dissolve or remove stubborn corrosion products without further harm, requiring expertise to avoid damaging the artifact. Protective coatings create a barrier against moisture and corrosive agents, with the option of temporary or permanent applications depending on the artifact’s conservation needs. The choice of conservation technique depends on the artifact’s condition, corrosion type, metal composition, and intended use. In museum displays, more invasive methods may be necessary to restore appearance and stability, whereas storage in controlled environments may only need less invasive approaches. Preventive measures, such as maintaining a dry, stable environment and controlling temperature and humidity, can also protect buried leaded bronze artifacts. For ternary bronzes with high lead content, the deterioration process may involve the formation and crystallization of salts like lead (II) oxide (massicot, PbO) and carbonates (cerussite, PbCO_3_). The study found that the most effective treatment for bronze artifacts was using benzotriazole 2% dissolved in ethanol, followed by two layers of paraloid 2% in ethanol for final protection. Raman spectra analysis provided valuable insights into the corrosion products present in the patina cross-section, notably identifying brochantite (Cu_4_(SO_4_)(OH)_6_) with specific bands at 1110 cm^−1^, 300 cm^−1^, and 245 cm^−1^. The coexistence of brochantite and antlerite suggests that sulfur pollutants from organic matter decomposition by sulfate-reducing bacteria in the soil influence the formation and preservation of these corrosion products. X-ray diffraction (XRD) analysis of soil samples surrounding buried bronze statuettes revealed mineralogical compositions significantly influencing corrosion processes, providing critical insights for developing effective preservation strategies. Additionally, pH measurements indicated varying soil acidity and alkalinity levels, crucial in determining corrosion rates and mechanisms, offering essential data for targeted preservation strategies. The comprehensive Raman spectroscopic analysis offers valuable information for understanding and preserving bronze artifacts in archaeological contexts. Further research on additional bronze artifacts can deepen our knowledge of patina formation processes and contribute to developing effective conservation strategies for these historical objects. By applying appropriate conservation techniques and preventive measures, we can ensure the preservation of these artifacts’ historical, cultural, and scientific value for future generations.

## Data Availability

The dataset(s) supporting the conclusions of this article is (are) available in the article.
